# Multiscale analyses reveal native-like lamellar bone repair and near perfect bone-contact with porous strontium-loaded bioactive glass

**DOI:** 10.1016/j.biomaterials.2019.03.035

**Published:** 2019-07

**Authors:** H. Autefage, F. Allen, H.M. Tang, C. Kallepitis, E. Gentleman, N. Reznikov, K. Nitiputri, A. Nommeots-Nomm, M.D. O'Donnell, C. Lange, B.M. Seidt, T.B. Kim, A.K. Solanki, F. Tallia, G. Young, P.D. Lee, B.F. Pierce, W. Wagermaier, P. Fratzl, A. Goodship, J.R. Jones, G. Blunn, M.M. Stevens

**Affiliations:** a Department of Materials, Imperial College London, London, SW7 2AZ, United Kingdom; b Department of Bioengineering, Imperial College London, London, SW7 2AZ, United Kingdom; c Institute of Biomedical Engineering, Imperial College London, London, SW7 2AZ, United Kingdom; d Institute of Orthopaedics and Musculoskeletal Science, University College London, London, WC1E 6BT, United Kingdom; e Centre for Craniofacial and Regenerative Biology, King's College London, London, SE1 9RT, United Kingdom; f Max Planck Institute of Colloids and Interfaces, Department of Biomaterials, Research Campus Golm, Potsdam, Germany; g Mechanical Engineering, University College London, Torrington Place, London, WC1E 7JE, United Kingdom; h School of Pharmacy and Biomedical Sciences, University of Portsmouth, PO1 2DT Portsmouth, United Kingdom

**Keywords:** 3D porous bioactive glass, Strontium-releasing materials, Raman spectroscopy, Critical-sized bone repair, FIB-SEM, SAXS

## Abstract

The efficient healing of critical-sized bone defects using synthetic biomaterial-based strategies is promising but remains challenging as it requires the development of biomaterials that combine a 3D porous architecture and a robust biological activity. Bioactive glasses (BGs) are attractive candidates as they stimulate a biological response that favors osteogenesis and vascularization, but amorphous 3D porous BGs are difficult to produce because conventional compositions crystallize during processing. Here, we rationally designed a porous, strontium-releasing, bioactive glass-based scaffold (pSrBG) whose composition was tailored to deliver strontium and whose properties were optimized to retain an amorphous phase, induce tissue infiltration and encourage bone formation. The hypothesis was that it would allow the repair of a critical-sized defect in an ovine model with newly-formed bone exhibiting physiological matrix composition and structural architecture. Histological and histomorphometric analyses combined with indentation testing showed pSrBG encouraged near perfect bone-to-material contact and the formation of well-organized lamellar bone. Analysis of bone quality by a combination of Raman spectral imaging, small-angle X-ray scattering, X-ray fluorescence and focused ion beam-scanning electron microscopy demonstrated that the repaired tissue was akin to that of normal, healthy bone, and incorporated small amounts of strontium in the newly formed bone mineral. These data show the potential of pSrBG to induce an efficient repair of critical-sized bone defects and establish the importance of thorough multi-scale characterization in assessing biomaterial outcomes in large animal models.

## 1 Introduction

Biomaterial-based approaches are an attractive alternative for the repair of substantial bone defects that do not undergo full endogenous repair and have given rise to a lucrative market in regenerative materials [1]. Synthetic inorganic materials including bioactive glasses (BGs) and calcium phosphate ceramics [[2], [3], [4]] are particularly attractive because they encourage bone bonding. BGs have the benefit that they can deliver active ions that alter cell responses and stimulate bone regeneration [[5], [6], [7]]. The original BG composition (Bioglass^®^ BG45S5) has been used in more than a million patients as a synthetic particulate bone substitute for the repair of small dental and osseous defects [2,8]. BG elicits a strong bone-to-material bond through the formation of a carbonated apatite (HCA) layer on its surface. Moreover, as the material degrades, it releases ions including calcium, phosphate, and soluble silica species, which stimulate cellular responses such as bone formation and vascularization [[5], [6], [7]]. BG's compositional versatility also allows for modulation of the material's characteristics, including its suitability for sintering and degradation profile. Following reports demonstrating the efficacy of the anti-osteoporosis drug strontium ranelate (SrRan) [9,10], strontium has been incorporated into BG for bone repair, as BG's amorphous nature allows it to deliver strontium at a sustained rate. In *in vitro* studies, strontium-substituted BGs increased the anabolic and anti-catabolic activity of osteoblasts and osteoclasts, respectively [[11], [12], [13]], and strontium-substituted materials showed enhanced bone formation and osteointegration *in vivo* [[14], [15], [16], [17], [18], [19], [20]].

Despite such promising findings, studies describing bone repair mediated by inorganic bone substitutes point towards the need for three-dimensional (3D) porous structures and to elicit robust biological activity. This is because an appropriate 3D architecture provides mechanical support and space for cell infiltration and neovascularization prior to new bone formation [21]. However, while porous 3D scaffolds based on 45S5 bioactive glass have been reported [[22], [23], [24], [25], [26], [27], [28], [29]], 3D BG porous scaffolds that retain the amorphous structure [[30], [31], [32]] have proven more difficult to produce because commercially available compositions crystallize during sintering [24,25,[27], [28], [29]], disrupting their ability to form the bone-bonding surface HCA layer and inhibiting the release of cell-stimulating ions. To overcome these limitations and explore the hypothesis that such design criteria will enhance the ingrowth of normal bone, we designed a strontium-containing BG (pSrBG) by tailoring its composition, so that the glass would release a therapeutic range of strontium and also possess a broadened sintering window (temperature difference between the glass transition temperature, which must be surpassed for sintering to occur, and the crystallization onset temperature), making it possible to produce an amorphous 3D porous scaffold. We then investigated its ability to regenerate bone in a critical-sized defect in an ovine model [33], comparing its performance to the commercially available clinical standard bioactive glass, BG45S5. We examined the quality of the newly-formed bone using a combination of materials-based characterization techniques including scanning electron microscopy (SEM) imaging of focused ion beam-milled sections (FIB-SEM), small-angle X-ray scattering (SAXS) and Raman spectroscopy. By analyzing the cell-material interface and examining the newly-formed bone's biochemical signature and structural organization, these techniques allowed us not only to reveal the potential of pSrBG to generate high quality, locally-competent bone but also highlighted the potential of detailed materials analyses in understanding materials-driven bone repair.

## 2 Materials and methods

### 2.1 Material synthesis and processing

Glasses were made via the melt-quenching route. Silica (99.8%, Tarmac Ltd. or High Purity, Prince Minerals, Stoke-on-Trent), calcium carbonate (all ≥ 98%, Sigma Aldrich, UK), magnesium oxide, sodium carbonate, calcium phosphate, strontium carbonate and potassium carbonate were mixed according to their molar percentage, melted (1200–1400 °C) and quenched in deionized water to produce a frit. The frit was dried at > 100 °C and was ground with a Jet Mill (Hosokawa Micron Ltd. Runcorn, UK). Particles were sized by sieving.

BG45S5 (46.1 mol% SiO_2_, 24.4 mol% Na_2_O, 26.9 mol% CaO and 2.6 mol% P_2_O_5_) had particle size of 0.1–1 mm.

For pSrBG processing, a glass composition of 44.5 mol% SiO_2_, 4 mol% Na_2_O, 4 mol% K_2_O, 4.5 mol% P_2_O_5_, 17.8 mol% CaO, 17.8 mol% SrO, 7.5 mol% MgO was used and porous scaffolds were made as previously described [34], with a glass slurry produced (using particles < 38 μm), foamed with a surfactant and gelled by *in situ* polymerization [34]. The polymer was then removed by thermal decomposition immediately prior to sintering. Briefly, the following process was used: 100 g of glass powder and 30 g of the monomer methacrylamide (Fluka, > 98%) were gently mixed and 15 g of the crosslinker *N,N*′-methylenebisacrylamide (>98%) was added under agitation. dH_2_O (90 ml) was added to act as the solvent and 10 drops of the dispersant Dispex were incorporated, followed by 0.5 ml of Triton X-100 (surfactant). To start the polymerization reaction, 2 ml of initiator (0.52 g/ml ammonium persulphate (>98%) in dH_2_O), followed by 20 ml of the catalyst TEMED (*N,N,N′,N*′-Tetramethylethylenediamine, 99%) were added and the system was vigorously agitated. Agitation was stopped immediately prior to gelation and the gelled foam was dried (125 °C for 10 h). The samples, cut into cubes, were then placed at 350 °C for 1 h, followed by sintering for 3 h at 690 °C. These scaffolds were used for material characterization by X-ray diffraction, X-ray fluorescence (XRF), mercury intrusion porosimetry and X-ray microtomography (μCT) analyses. *In vitro* and *in vivo* studies were conducted on the material whose production was up-scaled at a ratio 1:5. Instead of cutting the materials into cubes, the resulting up-scaled sintered pSrBG scaffolds were then ground using a Kek cone mixer (Kemutec, USA) to give granules of sizes 1–3 mm.

### 2.2 In vitro cell culture experiments

Cytotoxicity measurements were performed according to the ISO-10993-5 procedure, using MC3T3-E1 cells [35]. Liquid extracts from pSrBG and BG45S5 (n = 6) were obtained by incubating scaffolds in alpha-Minimum Essential Medium Eagle (alpha-MEM, Gibco) at 0.2 g/ml for 24 h at 37 °C. The extracts were passed through a 0.2 μm filter to remove particle debris and placed at 37 °C in a 5% CO_2_ incubator overnight. Extract media were supplemented with 5% (v/v) FBS, 50 μg/ml ascorbic acid and 1% (v/v) penicillin/streptomycin (P/S). MC3T3-E1 cells (ATCC) were seeded at 20,000 cells/cm^2^ and left to adhere for 24 h in complete growth medium (alpha-MEM + 5% (v/v) FBS + 50 μg/ml ascorbic acid + 1% (v/v) P/S) prior to incubation with the extract media for 24 or 72 h. Metabolic activity was measured by AlamarBlue^®^ assay (Life Technologies, UK) according to the manufacturer's instructions.

Bone marrow-derived human mesenchymal stem cells (hMSCs) (Promocell GmbH) were expanded in Promocell hMSC growth medium and used before passage 6. To evaluate the ability of primary osteoprogenitors to grow on pSrBG scaffolds, 20,000 hMSCs were seeded on pre-incubated (alpha-MEM for 24 h) scaffolds and cultured in growth medium (alpha-MEM with 10% (v/v) FBS, 50 μg/ml ascorbic acid and 1% (v/v) penicillin/streptomycin (P/S). After 7 days, samples were fixed in 4% (v/v) paraformaldehyde/PBS for 15 min and dehydrated using a series of ethanol solutions, followed by incubation in hexamethyldisilazane (Sigma). Samples were dried and sputter-coated with Au before SEM imaging (Jeol 6010).

### 2.3 In vivo bone repair assessment

Non-pregnant skeletally mature female sheep at least two years old were allocated randomly to treatment groups (1 defect per animal, *n* ≥ 5 per condition and time point). Additionally, three recently sacrificed sheep of the same criteria were used to prepare time zero samples with surplus test materials. The work was supervised by G. Blunn and A. Goodship and conducted under approval of and compliance with the UK Home Office requirements, Animals (Scientific Procedures) Act 1986, which included local ethical approval by the Royal Veterinary College ethics committee.

One 8 mm diameter cylindrical defect was made in the left medial femoral condyle per animal, using an electric drill with a stop to limit the defect depth to 15 mm. As the critical defect nature of this model has previously been assessed, to reduce the number of sacrificed animals, no empty defect control was performed in this study. The critical nature of the defect, originated from a previous study [36], is clearly shown in Fig. S1. For consistency, a flat drill bit was used to flatten the bottom of the defect. The synthetic bone grafts were mixed with blood from the defect on implantation (2:1 ratio of ovine blood to BG material), allowed to coagulate, and the quantity necessary to fill the defect was inserted and compacted using a spatula. Kirschner wires were inserted adjacent to the defect for radiographic identification. The animals were not supported or immobilized after surgery. All animals were checked for post-operative adverse effects or local reactions to the implants. Blood samples were collected at defined time points and strontium content was measured. The sheep were sacrificed at 6 weeks or 12 weeks postoperatively with 0.7 ml/kg 20% intravenous pentobarbital. The left medial condyles were retrieved using an oscillatory saw.

Bone repair was assessed at 6 or 12 weeks postoperatively by peripheral quantitative computed tomography (pQCT), indentation testing (Zwick Roell Z005 H-frame compression-tensile loading machine using a 4 mm^2^ circular indenter), and histology and histomorphometry (Toluidine Blue/Paragon staining). 200 μm thick sections of 12 week samples were further prepared for SEM, high spatial resolution SAXS/XRF, FIB-SEM of the interface between the newly deposited bone and scaffolds and Raman spectral imaging. Contrast has been adjusted in histology and SEM images for better visualization.

### 2.4 Small-angle X-ray scattering (SAXS) and X-ray fluorescence (XRF)

Polished 200 μm sections of the resin embedded bone samples were investigated with an environmental scanning electron microscope (ESEM) (FEI-Company, Oregon, USA) in low vacuum using backscattered electron (BSE) mode at a working distance of 10 mm. The electron beam energy was set to be 10 kV. A solid state detector (SSD) was used to measure the BSE signal from the sample surface.

For laboratory measurements (with low spatial resolution imaging), SAXS equipment was used, based on an X-ray generator (Bruker, AXS, Karlsruhe, Germany) with a rotating copper anode operating at 40 kV/100 mA, producing an X-ray beam with a wavelength of λ = 0.154 nm. The diameter of the beam at the sample was 200 μm and the sample detector distance was 620 mm [37].

The bone sections were mounted on a sample holder which could be moved automatically with a precision of 2 μm in the plane perpendicular to the incident beam. Data were collected with an area detector (Bruker) and corrected for background scattering. Prior to the scattering measurements an X-ray transmission image of the bone sample was produced by measuring the X-ray absorption of the sample using a diode to determine the positions for the SAXS experiments. After the SAXS measurement, scattering patterns were obtained representing the intensity distribution scattered around the primary beam which was transmitted through the bone sample for 1 h. The two-dimensional SAXS patterns were analyzed for mean mineral crystal thickness (T-parameter) and the degree of alignment of the mineral crystals (ρ-parameter) within each sample. High spatial resolution measurements were carried out at the synchrotron beam line μSpot at BESSY II (Helmholtz-Zentrum Berlin für Materialien und Energie, Germany). Synchrotron radiation was used to measure SAXS and the X-ray fluorescence (XRF) of calcium and strontium simultaneously with a beam diameter of 30 μm [38]. The sample to detector distance was 313 mm and the wavelength of the X-ray beam was 0.06888 nm. Calcium and strontium distribution maps were created using the XRF signal to define the regions of interest for the measurements. An energy sensitive detector (ASAS-SDD KETEK, Munich, Germany) with a 100-mm^2^ sensitive active area and 167.4 eV energy resolution was used for the XRF measurements, allowing for the determination of variations in strontium and calcium contents.

### 2.5 Focused ion beam-scanning electron microscopy

Cross-sections of trabecular bone tissue at the interface between the newly deposited bone and BG45S5 or pSrBG samples were prepared and imaged using a FEI Helios 600 Dual Beam system as follows. Samples were demineralized (in 0.5 M EDTA, 2% (v/v) PFA, CaCo, pH 7), stained with alcian blue (for proteoglycan stabilization), post-fixed with 4% (v/v) glutaraldehyde, stained with osmium tetroxide and thiocarbohydrazide (conductive staining) and sputter-coated with Au. Areas of interest were coated with a protective 0.5–1.0 μm Pt layer in two steps at the ion current 0.28 nA first and then 0.92 nA. Ion milling of cross-sections ∼60 μm wide and ∼20 μm deep were performed at 30 keV with a gradual reduction of the milling current from 21 nA to 2.8 nA. Imaging was performed using mixed secondary-back-scattered electron detection. Each tiled cross-section spans an area of about 50 × 12 μm (with 10 nm pixel size) and comprises between 6 and 18 partly overlapping frames. The orientation of each cross-section is perpendicular to the bone/grain boundary. Results are representative of ≥2 bone-material interface cross-sections from 3 independent sections per material-treated defect at 12 weeks.

### 2.6 Raman spectral imaging

For Raman spectral imaging a confocal Raman micro-spectroscope (alpha300R+, WITec, Ulm, Germany) was used. All experiments were performed using a 532 nm laser and a 10 × /0.3 NA microscope objective lens. Raman spectral images of ∼800 × 800 μm were produced with 2 μm spatial resolution at 0.5 s integration time and spectral range 0–3000 cm^−1^. The Control FOUR software was used for all data collection.

The procedure carried out on each dataset included a baseline correction, smoothing (Savitzky-Golay) and normalization. K-means clustering was used to spectrally identify the different regions within the samples. The selected bone characterization parameters were calculated using the integrated areas of selected bands. For the mineral-to-matrix ratio (MMR) the ∼960 cm^−1^ phosphate band was divided by the sum of the ∼854 cm^−1^ and ∼871 cm^−1^ proline and hydroxyproline bands. The mineral-to-carbonate ratio (MCR) was calculated using the ∼960 cm^−1^ phosphate band divided by the ∼1070 cm^−1^ carbonate band. For the mineral crystallinity the FWHM of the ∼960 cm^−1^ phosphate band was used [39]. The Project FOUR Plus software was used for all data processing. All spectra presented were intensity calibrated according to the National Institute Standard and Technology (NIST) material. Results are representative of ≥2 Raman spectral images from 3 independent sections of pSrBG- or BG45S5-treated defect at 12 weeks.

### 2.7 Statistical analysis

Statistical analyses were carried out using 2-way ANOVA analysis with a Bonferroni *post-hoc* test to compare the treatment groups and time points, unless stated otherwise. Differences with *p*-values lower than 0.05 were considered significant.

Details of the materials characterization, *in vivo* explant preparation and analyses, and SAXS parameter calculations are provided in the SI experimental procedures.

## 3 Results

### 3.1 pSrBG is a non-toxic, strontium-containing, amorphous, highly porous BG

**Fig. 1:**
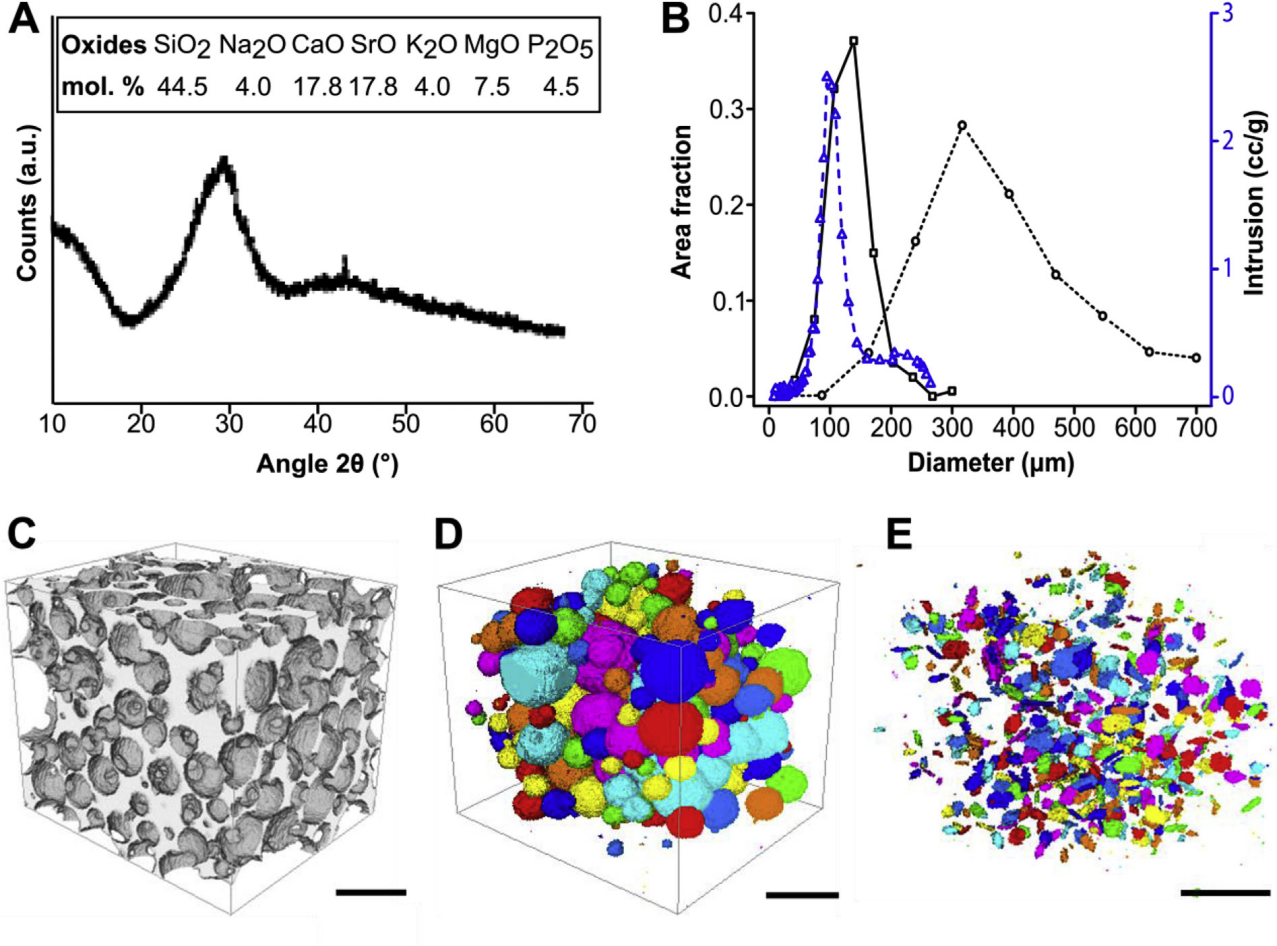
**pSrBG is a strontium-containing BG and displays highly interconnected pores. (A)** X-ray diffraction showing that pSrBG retains an amorphous phase and pSrBG composition, expressed in mol%. **(B)** Interconnect (solid black line) and pore (dotted black line) diameter distribution (measured by μCT), and interconnect diameter distribution measured by mercury porosity (dashed blue line). **(C**–**E)** μCT reconstruction of pSrBG scaffold showing (C) the whole scaffold, (D) the pores and (E) interconnects within the scaffold. Scale bars = 500 μm.

We have previously shown that other compositions of strontium-substituted BG upregulate osteoblast activity and downregulate osteoclast activity *in vitro* [11]. Moreover, using whole-genome microarray analysis, we showed that strontium-containing BG modulated human mesenchymal stem cells' (hMSCs) metabolic activity and triggered modifications in membrane composition [40]. Based on these results, we aimed to design a porous 3D BG scaffold that would allow a release of strontium ions in similar concentrations. Successfully formulating a 3D porous BG scaffold while preserving its amorphous phase, however, required us to finely tune its composition and processing parameters. Optimizing the composition was particularly critical, as it both defines the BG's biological activity and affords the material appropriate processing characteristics such as a wide sintering window. Using 45S5 composition as a basis for our design, the number of network modifiers (K^+^ and Mg^2+^), and the SiO_2_ content were slightly increased in order to broaden the sintering window. The total cation content with +1 charge was also reduced from 24.4 mol% Na_2_O in 45S5 to 12 mol% in pSrBG (6 mol% Na_2_O and 6 mol% K_2_O), to reduce initial rate of cation exchange and limit the increase in pH when scaffolds are immersed in fluid (Fig S2A). Strontium was included to reduce the relatively high Ca^2+^ content (35.6 mol%), and to play the role of a therapeutic agent as well as acting as another network modifier to further expand the sintering window. We determined that with 50 mol% of Ca^2+^ substituted by Sr^2+^, strontium ions were released *in vitro* at mM range concentration (Figs S2E and S3A) that is known to be susceptible to affect hMSC responses to BG *in vitro* [40]. Our optimized composition, which has been seen investigated with regards to dissolution and phase formation in aqueous solutions *in vitro* [41], consisted of 44.5 mol% SiO_2_, 4 mol% Na_2_O, 4 mol% K_2_O, 4.5 mol% P_2_O_5_, 17.8 mol% CaO, 17.8 mol% SrO, 7.5 mol% MgO (Fig. 1A and Table S1). We utilized a gel-cast foaming process, employing a combination of vigorous agitation in the presence of a surfactant and *in situ* polymerization [34] to create and maintain a porous structure. As anticipated, the pSrBG composition broadened the sintering window (174 °C compared to < 100 °C for BG45S5 [34]), allowing us to generate a BG that retained an amorphous phase: X-ray diffraction confirmed that, with the exception of a very small peak that could not be identified, sintered pSrBG scaffolds were predominantly composed of an amorphous phase (Fig. 1A; BG45S5 XRD pattern is shown for reference in Fig S4A). There was concern that the water based gel-cast foaming process may promote early onset crystallization at the particle surface, as seen in previous work foaming the ICIE 16 composition (49.46% SiO_2_, 36.27% CaO, 6.6% Na_2_O, 1.07% P_2_O_5_ and 6.6% K_2_O, in mol%) [34], but the XRD pattern (Fig. 1A) showed that this was not the case for pSrBG. MicroCT analyses showed that pSrBG had a homogeneous distribution of highly interconnected pores (Fig. 1B–E) that were likely suitable for cell infiltration, bone formation and vascularization [21]. We measured >3 interconnects per pore, a modal pore size >400 μm, and a modal interconnect size >100 μm (Fig. 1B). Mercury porosimetry measurements (Fig. 1B) and SEM on pSrBG cross-sections (Fig. S4B) further supported the presence of interconnects within the scaffold. Taken together, these results confirmed that pSrBG had many ideal physical properties for promoting bone repair.

To confirm that our designed composition would not elicit an acute cytotoxic response, we placed extracts containing ions dissolved from the material on mouse MC3T3-E1 cells, according to ISO-10993-5 (Fig. S3A–B). We saw no significant differences in cell metabolic activity between cells treated with pSrBG and BG45S5, confirming that pSrBG composition performed at least as well as standard BGs. Moreover, when hMSCs were seeded directly onto pSrBG, SEM images showed cells attached to the scaffold's inner and outer surfaces (Fig. S3C–D), suggesting that pSrBG was not only non-toxic, but could also support cell invasion and growth.

### 3.2 pSrBG is highly osteoconductive and encourages an effective bone repair

As our *in vitro* characterizations suggested that pSrBG was a good candidate for bone repair, we evaluated its ability to repair bone *in vivo*. To our knowledge, no study has yet evaluated the efficacy of a strontium-containing 3D porous bioactive glass to repair a critical-sized bone defect in a large animal model. We implanted pSrBG scaffolds, in the form of 1–3 mm granules, into critical-sized femoral condyle defects in sheep (drill hole, 8 mm diameter x 15 mm depth) and evaluated bone formation after 6 and 12 weeks. To assess the performances of our pSrBG scaffold in an *in vivo* pre-clinical setting, as a control, we used BG45S5 particles (0.1–1 mm), a commercially available, clinical standard BG product that cannot be formulated as a 3D amorphous scaffold due to its crystallization during sintering.

**Fig. 2:**
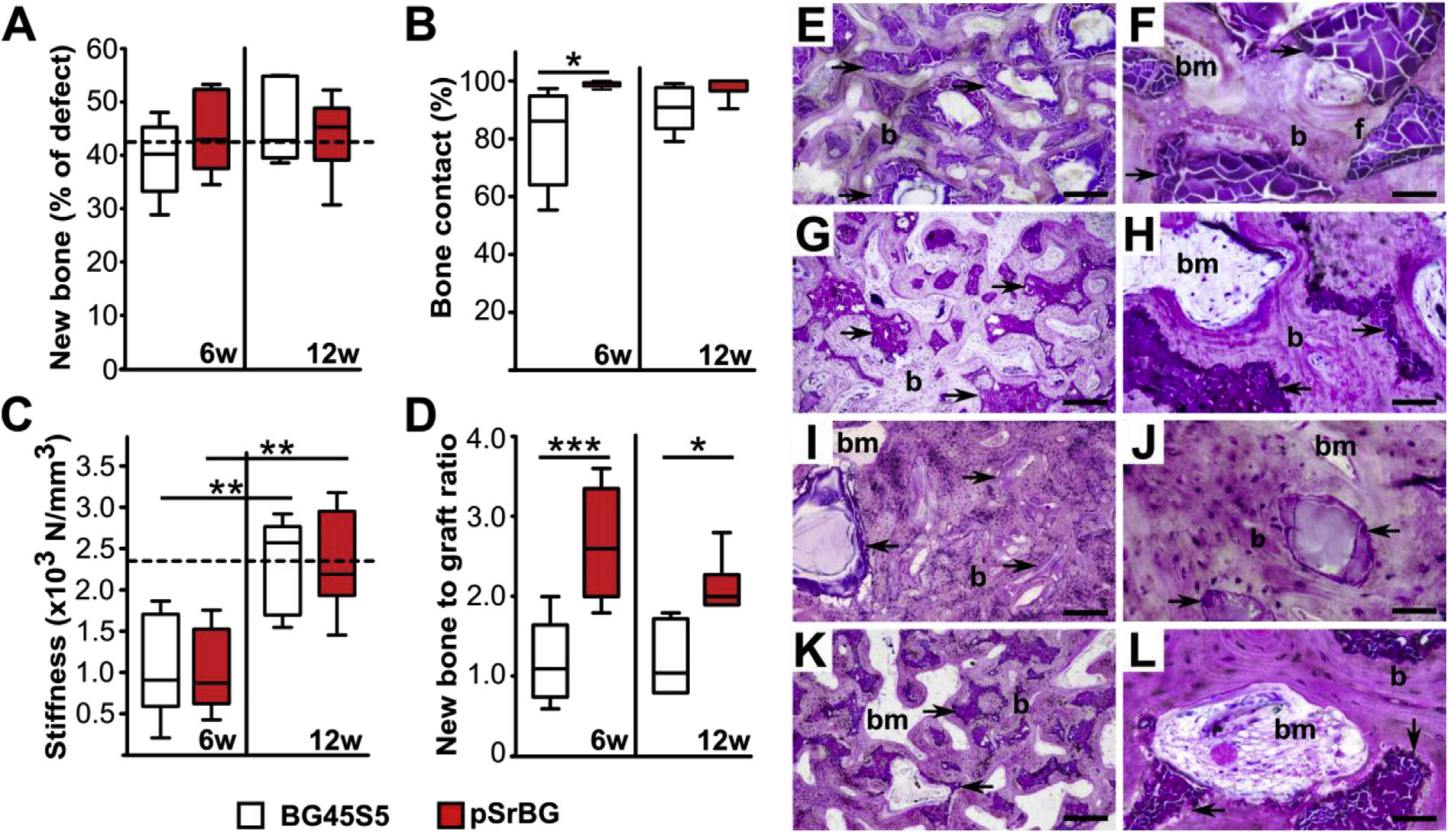
pSrBG shows enhanced osteoconductive properties and induces the formation of well-organized locally competent lamellar bone tissue. (A) Percentage of newly formed bone in defects treated with BG45S5 particles or pSrBG granules for 6 weeks and 12 weeks. The dashed line represents the median of the percentage of bone in non-defect areas (*n* = 19). (B) Percentage of bone that contacts the remaining scaffold. (C) Local mechanical assessment of the defect sites performed by indentation testing. The dashed line represents the stiffness median of the control trabecular bone in BG45S5-treated animals at 12 weeks (*n* = 5). (D) Ratio of newly-formed bone to the remaining scaffold. The box plots represent the 5th to 95th percentiles. Asterisks denote statistically significant differences between indicated groups (*p < 0.05, **p < 0.01, ***p < 0.001). In (A, B and D), at 6 weeks *n* = 5 and at 12 weeks *n* = 6, in (C) *n* = 6 for both time points. (E–H) Histological examination after 6 weeks of implantation of BG45S5 (E, F) and pSrBG (G, H). (I–L) Histological examination after 12 weeks of implantation of BG45S5 (I, J) and pSrBG (K, L). Histology sections were stained with Toluidine Blue and Paragon. Arrows point at the remaining synthetic materials; b represents new formed bone; bm represents bone marrow; f represents fibrous tissue. Scale bars in E, G, I, K are 300 μm; scale bars in F, H, J, L are 60 μm. (For interpretation of the references to colour in this figure legend, the reader is referred to the Web version of this article.)

We observed no adverse post-operative effects or local reactions to the implants. Initial histomorphometry analyses showed that after 6 weeks, both pSrBG and BG45S5 scaffolds allowed for bone growth into the defect. The percentage of newly deposited bone in both groups was consistent with that in normal femoral trabecular bone, measured in control areas away from the defect zones, with median values of 43%, 40%, and 42% for pSrBG, BG45S5, and control areas of normal trabecular bone, respectively. 12 weeks post-operatively, these values remained stable and we found no statistical differences at either of the time points we examined (Fig. 2A and Fig. S5A), confirming previous reports of the efficacy of BGs in promoting bone repair [6]. However, despite these similarities, pSrBG significantly outperformed BG45S5 in terms of scaffold bone coverage (*p* < 0.05 at 6 weeks). pSrBG scaffolds were remarkably 99% and 100% covered with new bone at 6 and 12 weeks (Fig. 2B), respectively, including internal pore walls. BG45S5, on the other hand, only showed 86% and 91% coverage, respectively. Moreover, while pSrBG-treated defects showed similar infiltration by soft tissue (i.e. bone marrow and blood vessels, 33%) when compared to the surrounding trabecular bone (58%) (Fig. S5B), BG45S5-treated defects were significantly more densely packed with bone and remaining synthetic material (14% soft tissue at 12 weeks, *p* < 0.05). This finding suggested a beneficial effect of pSrBG porous architecture on the structure of the newly formed bone, which was found to be more akin to normal trabecular bone in pSrBG-treated defects than that in defects treated with BG45S5.

As BG45S5-treated defects were more densely packed than those treated with pSrBG, we hypothesized that the BG45S5 repair tissue would be stiffer. To assess this, we performed indentation testing. Although the stiffness of the repaired tissue in both groups increased between 6 and 12 weeks, we could not detect significant differences between them and both were comparable to control regions of normal trabecular bone (Fig. 2C). This was particularly surprising as pSrBG-treated defects had less remaining implant material in the defect site than BG45S5-treated defects (*p* < 0.01 at both time points) (Fig. S5C). Thus the repair in pSrBG was mediated by a significantly higher newly formed bone-to-scaffold ratio than that in BG45S5 (Fig. 2D). Taken together, these data suggest that while BG45S5 formed a dense repair tissue supported by stable synthetic material, pSrBG elicited the formation of trabecular bone with strength, density and a soft tissue composition similar to that of normal bone. In short, pSrBG was highly osteoconductive and promoted an effective repair with local properties similar to that of native trabecular bone.

### 3.3 pSrBG promotes the formation of well-organized lamellar neo-bone tissue

We also carried out histological analyses to macroscopically characterize the newly-formed bone in the defects (Fig. 2E–L). Our analyses confirmed the presence of new bone in both pSrBG- and BG45S5-treated defects, marked by the presence of osteocytes, lacunae, bone marrow, and blood vessels. Such features were present 6 weeks after implantation and were more abundant after 12 weeks. Nevertheless, differences between the BG45S5 and pSrBG scaffolds were again apparent. We corroborated our exciting previous observation of 100% bone-to-material contact, characterized by a complete lack of fibrous tissue coverage (Fig. 2 G, H, K, L) on pSrBG scaffolds only 6 weeks after implantation, while at this time point, fibrous tissue could clearly be observed adherent to the surface of BG45S5 (Fig. 2F). Moreover, whereas overall, defects treated with BG45S5 were compact and composed of newly-formed bone surrounding randomly-dispersed glass particles (Fig. 2 E, F, I, J), pSrBG-treated defects contained well-organized lamellar bone characterized by bone marrow and blood vessels within scaffold pores (Fig. 2 G, H, K, L). These histology observations suggested that, while the possibility of being partly affected by the grafts' arrangement within the defects during implantation existed, the differences in histomorphometry parameters found between pSrBG and BG45S5 were primarily the result of the materials’ physico-chemical characteristics (i.e. porous architecture and composition).

SEM backscattering images confirmed histology findings and showed mineralization of the newly-formed bone tissue (Fig. S6). These data demonstrate pSrBG's ability to promote robust bone formation, and importantly, bone formation directly at the implant surface.

### 3.4 pSrBG promotes deposition of lamellar neo-bone at the bone/material interface by 12 weeks

**Fig. 3:**
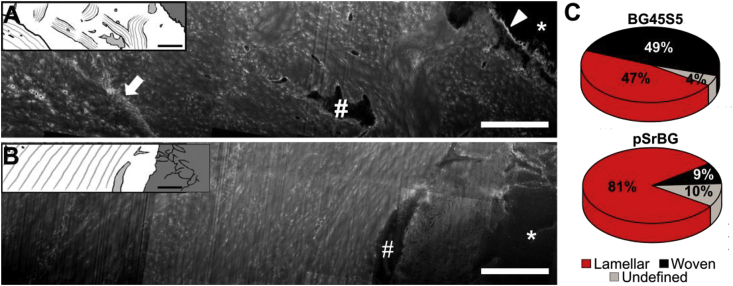
**FIB-SEM investigations reveal that pSrBG promotes lamellar neo-bone at the bone/material interface at 12 weeks.** (**A-B**) Broad cross-sections (compound images) through the interface between the implant particle on the right hand side and the newly deposited bone. **(A)** BG45S5 sample cross-section shows a broad intensively stained scalloped boundary (arrow head) that separates the particles from bone containing chaotically oriented bundles. An irregularly shaped osteocyte lacuna can be observed in this region (#). Another interface (arrow) separates this presumably woven bone from parallel arrays of collagen, characteristic for lamellar bone. In pSrBG **(B)**, collagen fibrils are deposited directly on the granule surface with no obvious non-collagenous interface. To the left of the osteocyte lacuna (#) bone matrix is organized in lamellar layers. Asterisks represent remaining inorganic materials. Graphics are inserted to facilitate visualization. **(C)** Percentage of woven and lamellar bone at the material interface in ion-milled cross-sections from three pSrBG- and BG45S5-treated defect samples (2 or 3 cross-sections per sample). The reader is invited to refer to Table S2 for more detailed information regarding the distribution within each sample. Scale bars are 5 μm.

These promising results from our histological analyses encouraged us to gain further insight into the quality of the repaired tissue and the bone/material interface. Therefore, we next examined bone quality, using a powerful combination of techniques. As bone mineral is templated on collagen, we examined collagen organization in the treated defects. We demineralized sections and utilized a powerful technique based on focused ion beam milling combined with scanning electron microscopy (FIB-SEM) to image the interface between the scaffold and newly formed tissue (Fig. 3) [42,43]. Sections from both the pSrBG- and 45S5BG-bone interface contained abundant collagen fibrils (recognizable by their D-periodicity), lacunae connected by canaliculi, and fragments of shrunken osteocyte vestiges. However, different features were observed in the newly-formed bone adjacent to pSrBG when compared to BG45S5. In BG45S5-treated defects, we often observed a <1 μm thick, intensely stained interface between the bone and implant, indicative of non-collagenous organic material between the implant and the newly-formed bone (Fig. 3A). We observed no such well-defined interface in pSrBG-treated defects. This tissue contained spindle-shaped osteocyte lacunae surrounded by electron-dense halos and alternating ∼3 μm thick lamellar bundles - features indicative of a canonical lamellar structure [42,43] (Fig. 3B). Conversely, at the interface with BG45S5, we observed irregularly-shaped osteocyte lacunae and a collagenous matrix containing 2–3 μm thick bundles of fibers with random orientations (Fig. 3A). This woven bone-like layer was delimitated from adjacent lamellar bone by an intensely stained sub-μm-thick line characteristic of a ‘cement line’: a ubiquitous finding at interfaces between old and new bone [44]. Quantitative analyses of several cross-sections (≥2 FIB-milled cross-sections from 3 independent samples per condition) showed that tissue adjacent to BG45S5 was composed of ∼49% woven bone, while 81% of the bone at the interface with pSrBG was lamellar (Fig. 3C, Table S2). In short, this micro-scale FIB-SEM analysis of the bone/material interface allowed us to uncover that while the BG45S5-bone interface was composed of both lamellar and woven bone, indicative of tissue repair, the bone-material interface mediated by pSrBG was almost exclusively lamellar as is found in normal, functional bone tissue, suggesting possible differences in bone formation or remodeling mechanism between the scaffolds.

### 3.5 pSrBG promotes native-like mineral nano-scale crystal characteristics in neo-bone

**Fig. 4:**
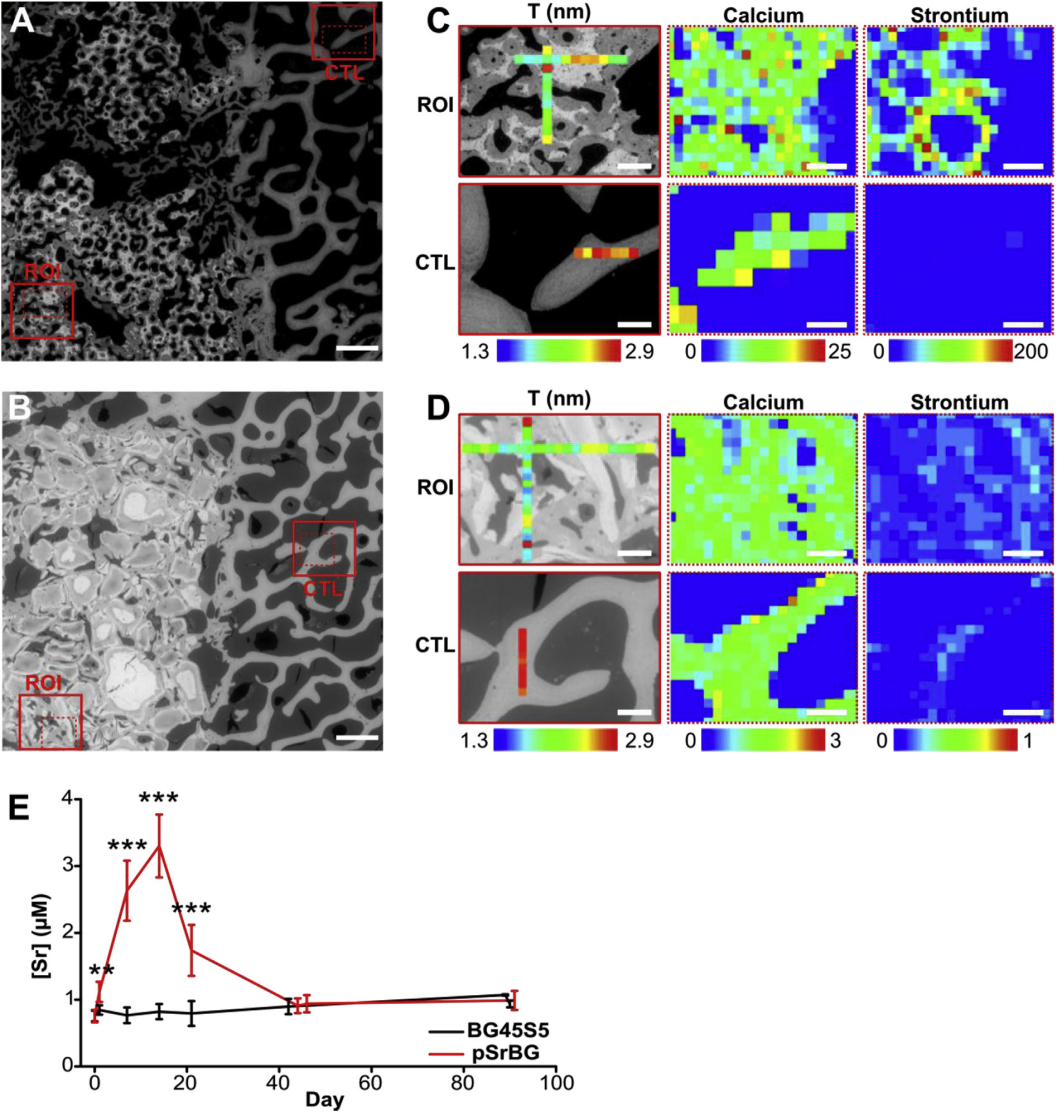
**pSrBG-released strontium incorporates into the neo-bone exclusively and promotes native-like bone mineral crystal thickness. (A**–**B)** ESEM image of (A) pSrBG- and (B) BG45S5- treated defect at 12 weeks showing the regions of interest (ROI) and control (CTL). **(C**–**D)** SAXS measurement of T-parameter (left) and XRF reading (right), showing the localization of strontium and calcium ions, in the defects treated with (C) pSrBG and (D) BG45S5. Scale bars in (A, B) are 1 mm; scale bars in (C, D) are 250 μm for T-parameter (left) and 200 μm for XRF reading (right). **(E)** Serum strontium concentration measured in the blood of the animals over 90 days. Data are expressed as mean ± SD. Asterisks denote statistically significant differences between indicated conditions (**p < 0.01, ***p < 0.001) as found by performing a Dunnett's Multiple Comparison Test with *t* = 0 as control (*n* = 12).

Studies in the 1930s indicated that animals fed large amounts of strontium developed a condition called ‘strontium rickets’ [45], which was characterized by abnormal bone mineralization. Although re-evaluation of these studies now attributes poor mineralization to calcium deficiency rather than strontium toxicity, concerns remain that replacing calcium with the larger strontium atom in the bone apatite lattice could produce mineralization defects. To examine this possibility, we performed SAXS measurements on sections taken from defects sites 12 weeks after implantation [46,47]. We measured ρ- (mineral crystal alignment) and T- (average thickness of the mineral crystals) parameters and found no significant differences between them (Fig. 4 and S7) indicating that strontium in the pSrBG scaffold did not appear to affect bone apatite crystal characteristics at the nano-scale. Interestingly, when we compared regions of interest in our treated defects to control regions for both samples, we found the T-parameter to be lower in the newly formed bone (the ρ-parameter was not significantly different) (Fig. 4 and S7), where mineral deposition was still ongoing.

### 3.6 pSrBG-released strontium incorporates exclusively in the neo-bone

While we could not detect an impact of strontium in pSrBG-treated groups on bone apatite crystal characteristics, we did find it in the animals' plasma up to day 21 (Fig. 4E), demonstrating that it was effectively released from the scaffolds for a sustained period of time. Nevertheless, the level of strontium we detected in the animals’ plasma was 40-fold lower than that measured in the plasma of patients treated with SrRan (0.12 mM), indicating that strontium can be released from a biomaterial without producing systemic effects [9]. Supporting this finding, pQCT analyses of the pSrBG-treated defects showed a decrease in density at 6 weeks, consistent with the loss of strontium from the scaffold (strontium has a higher X-ray density than calcium) [48] (Fig. S8). When we evaluated the spatial distribution of strontium in newly formed bone in pSrBG-treated defects by XRF, we found it co-localized with calcium, but absent from both control regions away from the defect (Fig. 4C) and BG45S5-treated defects (Fig. 4D). This finding confirms previous studies in SrRan-treated patients, which show that strontium incorporated only into newly formed bone [46]. XRF maps also highlighted that strontium remained in the pSrBG, suggesting that although strontium was released, the scaffolds remain a reservoir for potential further release.

### 3.7 pSrBG promotes neo-bone with a native-like biochemical signature

**Fig. 5:**
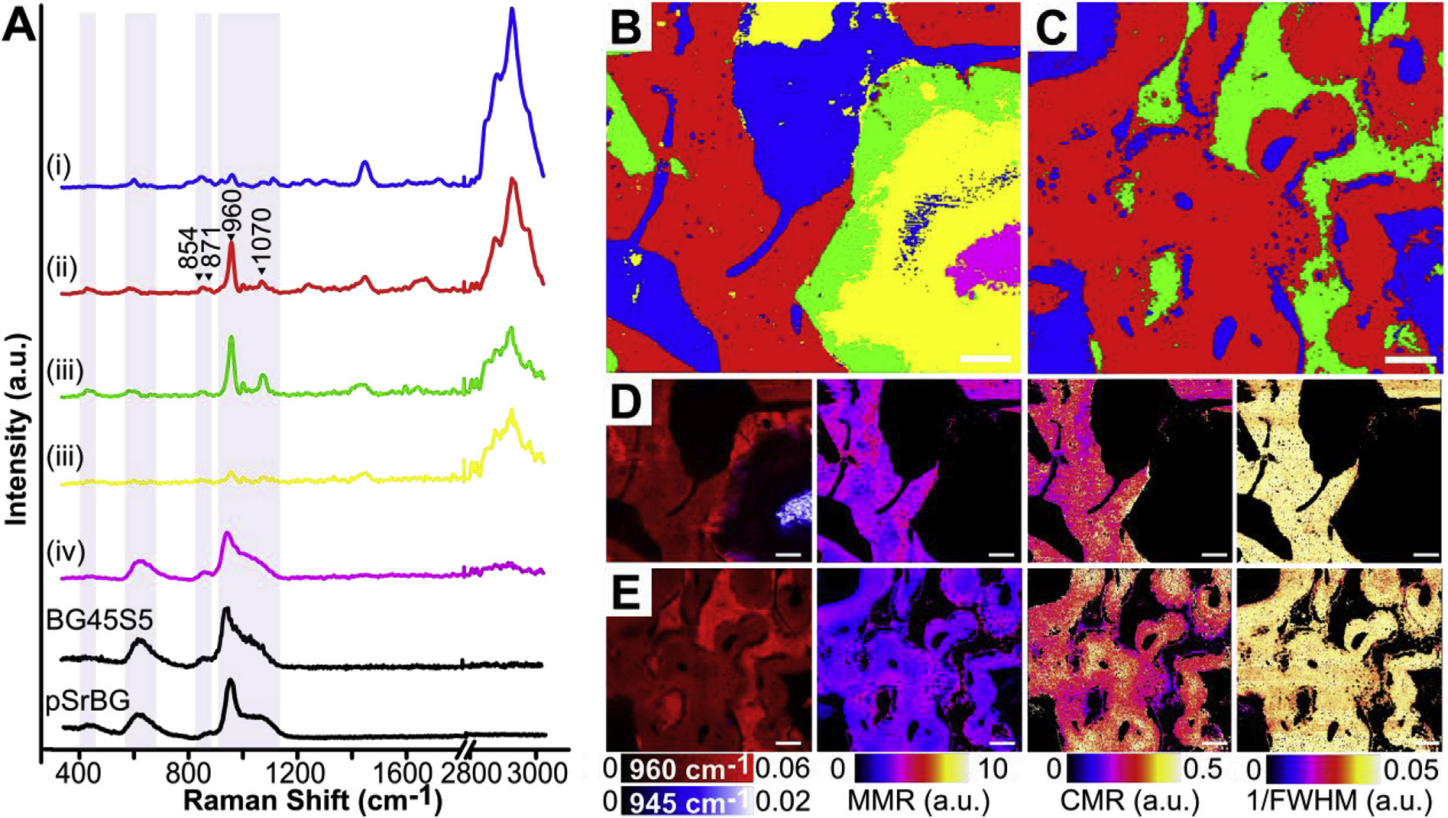
**pSrBG promotes neo-bone with a native-like biochemical signature.** Raman spectral imaging of (B, D) BG45S5- and (C, E) pSrBG- treated defects at 12 weeks. (**A**) Characteristic Raman spectral signatures, identified by k-means clustering analysis, representing the (i) soft tissue, (ii) bone, and (iii) synthetic material after modification in contact with biological fluids and (iv) synthetic material with similar characteristics to non-implanted BG45S5. As produced, pSrBG and BG45S5 are shown for reference (black lines). Spectroscopic BG modifications following implantation include a decrease in intensity of the bands attributed to the Si-O-Si groups (∼560-620 cm^−1^, 857 cm^−1^, 1000-1200 cm^−1^), a shift of the phosphate ν_1_P-O peak from ∼945 to ∼960 cm^−1^ and an increase of the ν_2_P-O band at ∼430 cm^−1^ (highlighted in grey). (**B-C**) Distribution of the characteristic spectra identified by k-means clustering analysis. (**D-E**) Heat maps (a.u.) of the 960 cm^−1^ and 945 cm^−1^ peaks, mineral-to-matrix ratio (MMR) (obtained by dividing the 960 cm^−1^ band and the sum of the ∼854 cm^−1^ and ∼871 cm^−1^ proline and hydroxyproline bands), carbonate-to-mineral ratio (CMR) (obtained by dividing the ν_1_CO_3_^2−^ band at 1070 cm^−1^ and the 960 cm^−1^ band) and, mineral crystallinity (1/full width half maximum (1/FWHM) of the 960 cm^−1^ peak). Scale bars are 100 μm.

We next examined if strontium's presence in the BG impacted established spectroscopic measurements of bone quality [39,49]. We carried out Raman spectral imaging on samples 12 weeks after implantation and used unsupervised classification analysis techniques to probe for changes in bone quality (Fig. 5). A k-means clustering analysis of Raman spectra collected from both BG45S5- and pSrBG-treated defects identified groups indicative of: (i) soft tissue, (ii) newly-formed bone, and (iii) synthetic material modified following contact with biological fluids [50] (Fig. 5A–C and S9A-D). This observation confirmed previous studies that show the presence of an interfacial region between BG and surrounding tissue through the precipitation of HCA [2,8], and is in line with the kinetics of dissolution/precipitation observed during immersion in simulated body fluid (SBF), indicating that both BG45S5 and pSrBG follow a similar mechanism of HCA formation to the one described by Hench (Fig. S2 and [51]). Interestingly, the Raman data further showed the inner portion of remaining large BG45S5 particles produced an additional cluster (iv) characteristic of the material as initially produced [50], while this feature was not observed in pSrBG-treated defects. The newly-formed bone in both BG45S5- and pSrBG-treated defects produced a single cluster with comparable quality, as measured by mineral-to-matrix ratio (MMR), carbonate-to-mineral peak ratio (CMR), and full width half maximum (FWHM) (Fig. 5D and E), indicating that there were no detectable differences in the bones' biochemical composition between the two treatments. Moreover, for both treatments, no statistical differences in these bone quality parameters, with the exception of FWHM (higher in both treatment groups *vs.* control), were found between the newly-formed bone and control region (Fig. S9) [39,52]. FWHM is often used as a measurement of bone crystallinity and lower FWHM is associated with aged bone. Therefore, these data suggest that the strontium in pSrBG had little effect on the quality of newly formed bone, and the only differences we could detect could be attributable to the abundance of newly formed, relatively immature bone in the pSrBG-treated defects.

## 4 Discussion

Here we developed a 3D porous strontium-containing BG using a gel-cast foaming method on an optimized BG composition. This process allowed for the formation of a porous BG with homogenously distributed, interconnected pores optimized for bone in-growth and blood vessel formation [21]. Gel-cast foaming requires sintering, which in the past often resulted in BG crystallization due to a small sintering window. Our optimized composition enlarged the sintering window and facilitated the material's design as a 3D porous scaffold that retained an amorphous phase, a feature that cannot be achieved using conventional BGs such as 45S5. To enhance its biological activity, we added strontium to harness its therapeutic properties [9,10]. Although strontium's mechanism of action remains unknown, strontium-containing inorganic bone substitutes increase bone formation and osteointegration *in vivo* [[14], [15], [16], [17], [18], [19], [20]], and modulate the activity of osteoprogenitors, osteoblasts, and osteoclasts, and macrophage phenotype *in vitro* [11,12,[53], [54], [55], [56]]. We have recently shown using a whole-genome microarray analysis that strontium incorporation in BG45S5 triggered significant changes in the global gene expression patterns of hMSCs and resulted in important changes in their metabolism and membrane composition [40]. Based on that evidence, we expected the incorporation of strontium into pSrBG to have a positive impact on the bioactive glass's osteoconductive potential. pSrBG also released other ions that have been shown to modulate cell responses, such as magnesium or silicate species (Fig. S2). Together with strontium, those ions would be expected to influence the material's biological activity and, as a result, bone formation [57,58]. Overall, pSrBG presents good biomaterial candidacy as it shows: (i) a 3D morphology with homogeneous pores of optimal size; (ii) an amorphous structure, essential for surface interactions and ion release; (iii) good *in vitro* biocompatibility; and (iv) strontium release, which can regulate osteoprogenitor biological activity.

While most functional studies focusing on evaluating the properties of strontium-containing bone substitutes are performed on rodents, we used a large animal model that reflects more of the human clinical situation. We evaluated the ability of pSrBG to regenerate bone in a critical-sized defect in an ovine model and compared it to the performance of the commercially available and clinical BG standard BG45S5. Histological and histomorphometry analyses showed that the amount of newly-deposited bone in defects was comparable between pSrBG, BG45S5 and normal healthy trabecular bone as early as 6 weeks post-implantation. Measurements of defect stiffness further showed that the newly-formed bone in both groups possessed similar local mechanical properties to that of control regions of trabecular bone. However, while fibrous tissue was present at the implant surface in BG45S5-treated defects, a result that was expected after only 6 weeks of implantation of a bone substitute, we remarkably observed near 100% bone contact with pSrBG, characterized by a quasi-total absence of fibrous tissue at the interface with pSrBG. Other synthetic bone substitutes do not usually elicit such a high percentage of bone-material contact in such a short period of time post-implantation. This result not only demonstrated that pSrBG's osteoconductive properties significantly outperformed those of BG45S5, but also raises the intriguing question whether such differences in newly-deposited tissue characteristics may be the result of the scaffold's influence on the bone regeneration/remodeling kinetics or mode of action.

Dissolution kinetics in SBF or buffers (*e.g*. Tris buffer) can provide important information to better characterize how the partial dissolution of the BG might change the implant local microenvironment with regards to ionic concentration and pH, and to assess the materials' ability to form an HCA layer at this surface [32,59,60]. Incubation of pSrBG and BG45S5 in SBF triggered, as expected, an initial quick release of cations and phosphate, followed by a decrease in calcium and phosphate contents from the SBF for both materials (Fig. S2). Those observations are in accordance with the mechanism of HCA formation described by Hench [51]. Interestingly, however, the rate of decrease of calcium and phosphate ions was higher when BG45S5 was immersed in the SBF than for pSrBG. This suggested the formation of HCA on BG45S5's surface was quicker than on pSrBG samples. As the cation release and the calcium phosphate deposition was more rapid for the BG45S5 powder compared to pSrBG powder of similar particle size, the difference in behavior was attributed to the glass composition rather than the morphology (Fig. S2). The materials' efficiency in forming the HCA layer at their surface is sometimes deemed to be correlated to the *in vivo* outcome of calcium phosphate-containing materials. While our findings indicated that both BGs have the potential to form an HCA layer at their surface layer, they did not show a direct correlation between the HCA formation and the *in vivo* higher bone-to-material contact observed in pSrBG samples.

We aimed to further assess the bone/material interface and characterize the newly-formed bone quality. Most studies investigating bone substitute performances focus on bone quantity and histomorphometry parameters but fail to account for the quality of the repaired tissue. However, in-depth assessment of bone quality has been demonstrated to be highly relevant for the characterization and investigation of bone pathologies and would largely benefit the bone tissue engineering/biomaterial community. The quality of bone tissue is dependent on a number of parameters including its macrostructure, collagen organization, degree of mineralization, and crystallinity. Raman spectral imaging, SAXS and FIB-SEM reconstruction are state-of-the-art techniques that have recently emerged as important tools to characterize normal and pathological bone quality [39,43,46,52]. Together, they allowed us to examine the structural organization of the newly formed bone, the bone-material interface, and the effect of strontium on bone biochemical and mineral characteristics. Our results show that pSrBG elicited the formation of more mature-like lamellar bone, similar to that in normal trabecular bone [61], when compared to that formed in BG45S5-treated defects. Moreover, as shown by FIB-SEM, newly-formed bone was in direct contact with pSrBG and possessed a lamellar organization. The bone-BG45S interface, on the other hand, was composed of both woven- and lamellar-like bone, which were delimited by a cement line, a feature of the classical bone remodeling process following repair [44]. These observations are enticing as they suggest that pSrBG, unlike BG45S5, may directly trigger the formation of well-organized lamellar bone.

When we evaluated the newly-formed bone in contact with pSrBG by Raman spectroscopy, we could detect no differences in markers of mineralization and bone maturity compared to healthy bone [39,49], other than a higher FWHM (indicative of lower crystallinity). Although these biochemical signatures are consistent with younger bone, such changes have also been described following strontium incorporation within the apatite lattice [62,63]. We carried out SAXS analysis to examine this possibility and detected a decrease in the average thickness of the bone mineral crystals and mineral crystal alignment when compared to control regions. However, we observed similar decreases in BG45S5-treated defects, suggesting that these observations are likely attributable to the relative bone tissue age [47] rather than the presence of strontium [46].

Our XRF analyses demonstrated the presence of strontium in newly-formed bone and in remaining material, but not in trabecular bone adjacent to the defect. These findings are consistent with those obtained from bone biopsies of SrRan patients and likely reflect the incorporation of strontium into bone apatite via substitution for calcium ions during bone formation [46,64,65]. The release of ions from pSrBG was further confirmed by pQCT analysis and the presence of strontium in the blood of the animals. Dissolution extracts from strontium-containing BGs have previously been shown to induce hemolysis *in vitro* at high concentration (>250 mg/ml) while lower concentrations did not trigger a significant hemolysis of red blood cells [52]. While the local concentration of strontium that can be reached at the implant site still remains unknown, we did not observe any adverse effects of pSrBG implantation on the animal health and as strontium was only detected in the circulating blood for 21 days following implantation and with a maximum concentration 40 times lower than that measured in the plasma of SrRan patients [43], the risk of potential systemic effects appears low. However, importantly, this also suggests that, locally, the change in ionic environment, including the presence of strontium, may be sustained for a period of time sufficient to trigger specific biological responses.

In conclusion, our study describes the potential of pSrBG to repair critical-sized bone defects and is an attractive alternative to autologous bone transplantations. There is a growing interest in synthetic inorganic porous scaffolds for bone repair. For example, clinical trials for spinal fusion have been recently published for Actifuse^®^, a granular silicon-containing porous hydroxyapatite. However, while promising, such studies show the challenge of producing inorganic scaffolds that can effectively compete with current clinical standards [66]. Here, our design of pSrBG fulfills many requirements [2] as it allows for cell in-growth and matrix deposition while ensuring mechanical support and progressive degradation as new bone forms. When implanted in the same animal model that we used here, Actifuse^®^ elicited a ‘medium-to-high’ bone coverage on the scaffold [3]. We observed nearly 100% bone-to-implant contact on pSrBG scaffolds, which highlights the clinical potential of pSrBG. Whether this resulted from enhanced osteoconductive properties or an osteoinductive effect, and what the contribution is of pSrBG's composition *vs.* its structural characteristics, such as its macro- and micro-porosities and surface roughness, and its packing density within the defect [3,4] remains difficult to determine. With the lack of commercially available (gold-standard) amorphous BG controls with similar porous structure to pSrBG, it remains challenging to investigate independently the influence of each of these parameters on the results obtained. However, it is evident that both composition and porosity are expected to play an important role in the scaffold tissue regeneration properties, as well as in the material's fate (e.g. dissolution kinetics, etc.), further emphasizing the benefit of producing amorphous BGs as 3D porous scaffolds that are able to modify their local ionic environment by releasing ions with therapeutic properties upon implantation.

Besides describing the development and high osteoconductive properties of pSrBG in a large animal model, this work also demonstrated the benefit of performing a thorough multi-scale analysis of the bone/material interface. Our comprehensive investigation not only showed that pSrBG allowed for the regeneration of high quality bone and had a very small risk of systemic effects, but it also provided information regarding strontium release kinetics and localization, and highlighted differences between the two implanted scaffolds in terms of material's fate and bone/material interface, showing a strong influence of the scaffold type on the characteristics of the tissue that is newly-deposited at the implant surface. These findings support the relevance of investigating the bone material/interface and the tissue and material physico-chemical characteristics when assessing material outcomes, especially when considering inorganic materials that dynamically interact with the local microenvironment, resulting in partial dissolution, modification of local ionic concentrations and important and rapid changes in materials surface properties.

In summary, we have developed a 3D porous strontium-containing bioactive glass that retains its amorphous phase and allows a sustained release of strontium *in vitro* and *in vivo*, and demonstrated it outperformed BG45S5 with regards to bone-to-material contact in a critical sized-defect in a large animal model. Our in-depth analysis of the bone and material characteristics following implantation showed the newly-formed bone displayed a physiological matrix composition that contained a small amount of strontium, and identified unexpected differences in bone structural architecture at the macro- and micro-scales between BG45S5 and pSrBG, with pSrBG promoting the formation of a more mature-like lamellar bone, rather than woven bone. This study shows pSrBG's potential as a future treatment for clinically difficult bone defects and demonstrates the utility of adopting a thorough multiscale materials-based characterization approach when investigating bone substitute performances.

## Author contributions

H.A., J.R.J., G.B., A.G. and, M.M.S. designed research; J.R.J., M.D.O. and, M.M.S. designed the material; H.A., F.A., C.K., K.N., E.G., N.R., A.G., G.B. H.M.T., A.N.N., P.D.L., C.L., B.M.S., W.W. and, P.F., T.B.K., F.T., and G.Y. performed research; M.D.O. and A.K.S. contributed to reagents; H.A., F.A., H.M.T., C.K., E.G., N.R., K.N, A.N.N., M.D.O., C.L., B.M.S., T.B.K., P.D.L., B.F.P., W.W., P.F., J.R.J., A.G. and, M.M.S. analyzed data; H.A., E.G., B.F.P., and M.M.S. wrote the original version of the paper; and H.A., J.R.J., and M.M.S. reviewed and edited the manuscript.

## Conflicts of interest

M.M.S. is a coinventor on a patent on strontium-containing BG (WO2007/144662).
