# Supplementary material for: Multiscale analyses reveal native-like lamellar bone repair and near perfect bone-contact with porous strontium-loaded bioactive glass
Source: Biomaterials. 2019 Jul;209:152–62. doi: 10.1016/j.biomaterials.2019.03.035 (PMC6527862; doi:10.1016/j.biomaterials.2019.03.035)
Supplement: Multimedia component 1 [file mmc1.pdf]

Multiscale analyses reveal native-like lamellar bone repair and near perfect bone-contact with porous strontium-loaded bioactive glass

H. Autefage[1,2,3], F. Allen[4], H.M. Tang[1], C. Kallepitis[1,2,3], E. Gentleman[5], N. Reznikov[1,2,3], K. Nitiputri[1,2,3], A. Nommeots-Nomm[1], M.D. O'Donnell[1], C. Lange[6], B.M. Seidt[6], T.B. Kim[1], A.K. Solanki[1,2,3], F. Tallia[1], G. Young[1], P.D. Lee[1,7], B.F. Pierce[1,2,3], W. Wagermaier[6], P. Fratzl[6], A. Goodship[4], J.R. Jones[1], G. Blunn[4*], M.M. Stevens[1,2,3*]

Supplementary information

SI Experimental Procedures

pSrBG material characterization

XRF analyses were performed on the glass powder used to produce pSrBG scaffolds and on the 3D porous material (Lucideon Ltd., Stoke-on-Trent, UK), using method C201 based on the BSEN ISO 12677:2011 procedure.

For micro-computed tomography (μCT) analysis, pSrBG samples were scanned using nano-focus μCT machine (Phoenix|X-ray General Electric Company Measurement and Control, Wunstorf, Germany) at a spatial resolution of 9 μm/voxel to provide pore and strut size information. Scan conditions were: 2000 ms acquisition time, 85 kV source voltage, 111 μA current, 1000 projections, 0.5 mm copper filter, reconstruction via filtered back projection (Datos x rec, Phoenix|X-ray General Electric). Accessible volume was used to quantify the strut and pore size distributions [1].

For X-ray diffraction experiments, pSrBG scaffolds were ground into a fine powder to be analysed and mounted onto a zero background single crystal silica substrate. The spectra were collected using a Bruker D2 PHASER desktop X-ray diffractometer with a step size of 0.0345°, 10 s per step, measuring between 5 and 70 degrees 2θ. The radiation source used was a Ni filtered CuKα.

Mercury intrusion porosimetry was carried out using a Quantachrome Poremaster 33 equipped with both high and low pressure functionality and fitted to a Thermo Renaissance 2005 ARL-LS vacuum pump. Due to the size of the interconnects within these scaffolds only low pressure analysis was completed. Five samples were prepared as cylinders of approximately 1 cm^3 and their masses and volumes were recorded. Samples were left to degas for 40 minutes prior to infiltration, the cells were then filled with mercury under nitrogen gas (pressure 340 kPa) and the pressure was recorded. The raw data was analysed using PoreMaster for windows 8.1.

Ion content following immersion in simulated body fluid

Simulated body fluid was prepared as previously described [2] and pSrBG scaffolds, BG45S5 particles, as well as their pSrBG and BG45S5 powders (< 38 μm) were incubated in the SBF at a ratio of 1.5 mg/ml (in 100 ml) [3]. Samples were agitated at 120 rpm using an orbital incubator at 37°C. After 1 hr, 2 hrs, 4 hrs, 8 hrs, 24 hrs, 48 hrs and 72 hrs of BG incubation, the pH of the solution was measured, and 2 ml of the solutions were taken and filtered through a 0.22 μm filter to ensure complete removal of potential BG material from the aliquots. 2 ml of stock SBF solution was added back to the flask to keep the volume constant. Samples were kept at 4°C before use. Elemental analysis of the solutions was performed using inductively-coupled plasma optimal emission spectrometry (ICP-OES iCAP6000 Series ICP spectrometer, Thermo Scientific) on samples diluted with a ratio 1:10 in filtered milliQ water. Standard solutions were prepared for each element within a range of 1 ppm to 40 ppm.

Sample preparation and analyses

The condyles were radiographed (to facilitate sectioning along the defect) and peripheral quantitative computed tomography (pQCT) scans were carried out to determine X-ray mineral density in regions of interest within the defect and in areas of cancellous bone adjacent to the defect. After scanning, the defect was sectioned in the frontal plane using an Exakt diamond-edged bandsaw. The cut surface of one half was subjected to indentation testing in a Zwick Roell Z005 H-frame compression-tensile loading machine using a 4 mm^2 circular indenter. Two aspects within the defect were tested to give a mean value of defect stiffness. Indentation testing was also carried out on control trabecular bone regions. The linear portion of load-deformation plots was used to calculate stiffness.

The other half of the sample was fixed, dehydrated and embedded in resin. The resin block was sectioned in the frontal plane and processed through using the Exakt technique.

Sections (95-100 µm thick) were stained with Toluidine Blue for 30 minutes (soft tissue) and Paragon for 20 minutes (bone) for histological evaluation and histomorphometric analyses.

For histomorphometric analyses, each section was divided into nine aspects to obtain the mean representation of the section. The percentage of bony ingrowth was measured using a Weibel grid composed of 225 points, from which, by measurement of total defect area and total area occupied by bone, the percentage of bone ingrowth was calculated. The bone coverage over the implant surfaces was measured from one aspect of the section and calculated as the percentage of material surface occupied by bone ingrowth. Sections of 200 µm thickness were further prepared for SEM, SAXS/XRF, FIB-SEM and Raman spectroscopy analyses.

T- and ρ-parameter calculations from SAXS data

To calculate the T-parameter, the SAXS data were radially integrated by a mathematical procedure based on Porod's law, using the specially designed software AutoFit (custom-made software, MPIKG). This procedure was described in detail in earlier works [4], [5]. The T-parameter is defined as $T = 4\Phi(1- \Phi)/\sigma$, where Φ describes the volume fraction of mineral in the bone tissue and σ represents the total surface of the mineral particles per unit volume. Thus the T-parameter measures the ratio of volume to surface of the mineral particles, characterizing their smallest dimension. As mineral particles in the bone are supposed to be thin platelets, T is a measure for the average thickness of the mineral particles.

The degree of mineral particle alignment (ρ-parameter) could be determined after azimuthal integration of the SAXS data [5], [6]. Depending on the orientation of the mineral particles within the plane perpendicular to the primary beam, the scattering patterns differ qualitatively. If the mineral platelets are aligned and perfectly parallel, the pattern shows a narrow streak perpendicular to the long axis of the mineral particles. If the particles are

oriented randomly, the SAXS pattern is spherical. Elliptical SAXS patterns are due to partially aligned mineral crystals, where the ρ-parameter describes the degree of alignment of these crystals. The intensity distribution, I(χ), shows two peaks separated by 180 °. The total area under the I(χ) curve is the sum of the area under the peaks, which is proportional to the fraction of parallel aligned particles, and the area under the constant background, which is proportional to the fraction of randomly oriented particles. The ρ-parameter is defined as the ratio (area under the peaks)/(total area). $\rho = 0$ indicates no predominant orientation of the mineral particles and $\rho = 1$ indicates perfectly parallel alignment.

Supplementary Figures

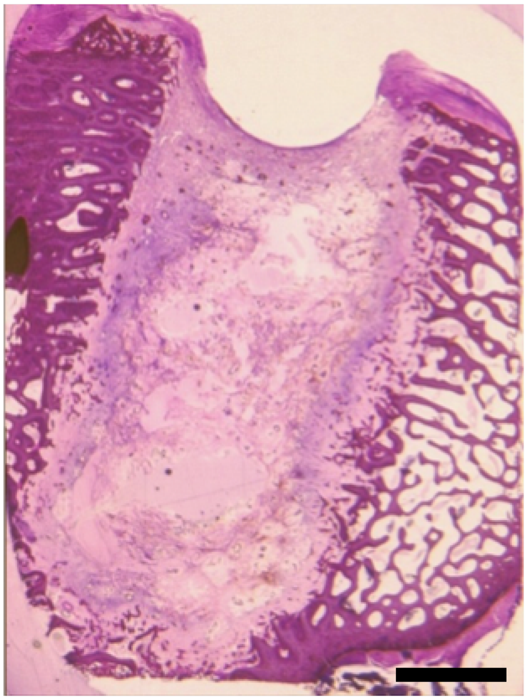

Figure S1. Histological section of an empty defect control. Histological evaluation of an empty defect at 12 weeks showed no sign of bone regeneration confirming the critical nature of the defect. Image supplied by Prof. G. Blunn, University College London and originated from a previous study [7]. Scale bar is 3 mm.

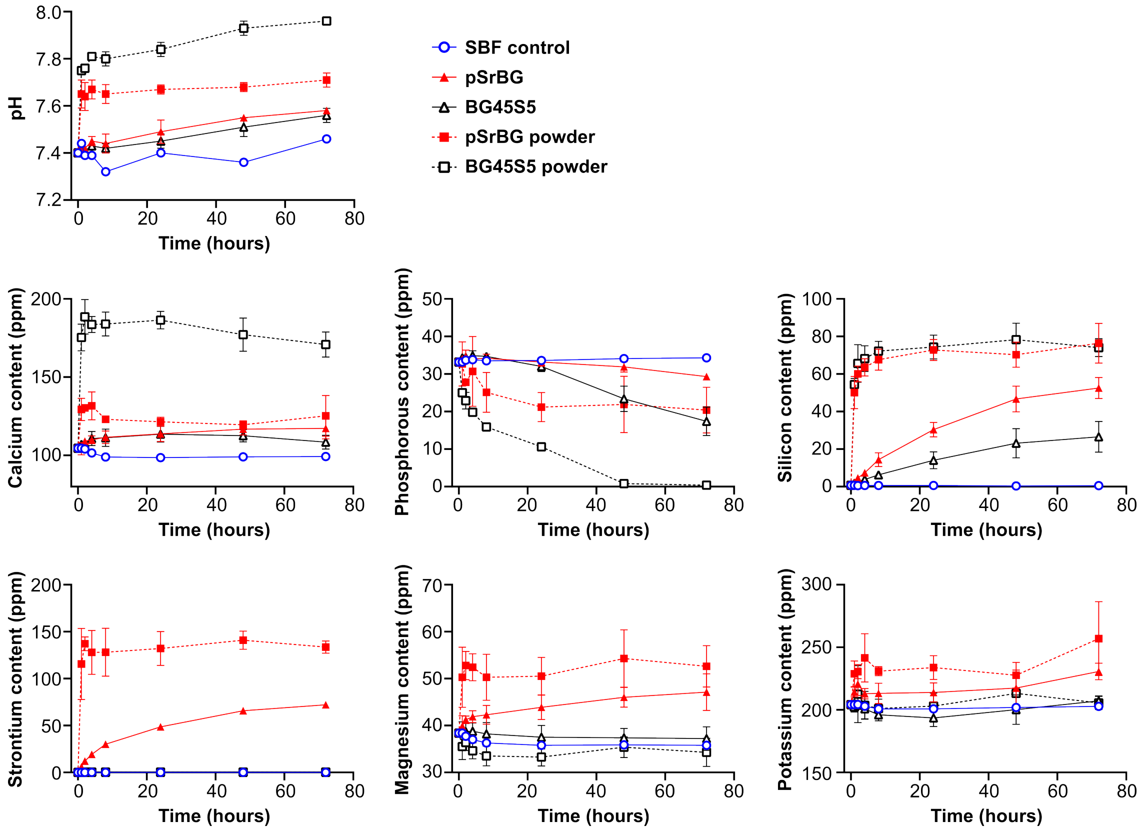

Figure S2: Results from the immersion of pSrBG granules and BG45S5 particles (same size as implanted *in vivo*) and pSrBG and BG45S5 powders (< 38 μm) in SBF at 37°C. (A) pH; (B-G) elemental concentrations of SBF measured by ICP-OES. Results are expressed as mean ± SD (n = 5 for pSrBG and BG45S5 scaffolds and n = 3 for pSrBG and BG45S5 powders).

As expected, the pH progressively increased as the BGs underwent dissolution in the SBF, but the increase was less pronounced for the pSrBG samples than for BG45S5, even when particle size was similar. Release increased as particle size decreased (surface area increased). ICP-OES measurements showed a rapid initial release of calcium, strontium, phosphate, potassium, magnesium, and silicon for pSrBG and calcium, phosphate and silicon for BG45S5, consistent with their respective composition. BG45S5 and pSrBG, when incubated as powders, both released cations and plateaued very quickly (within the first 2 hrs). Similar general profiles were found for the millimeter-sized samples, however the ions were released more slowly and the ion concentrations remained lower after 72 hrs of incubation. Following an initial increase, phosphorous and calcium levels were found to decrease with time for all materials. This feature is characteristic of the precipitation of a calcium phosphate layer (*e.g.* HCA) on the BGs' surface. According to the calcium and phosphorous ion contents, this precipitation was found to be quicker for BG45S5 than for pSrBG, which is likely due to the larger amount of calcium present in and released from BG45S5, and an inhibitory effect of strontium. Since this difference in precipitation kinetics was observed for both the powders and granules/particles, we can deduce that this is the composition rather than the materials' formulation process that plays a role in this event, as previously discussed elsewhere [8]. All together those results show that both pSrBG and BG45S5 displayed kinetics of ion release and precipitation that are in accordance with the mechanism of biomineralisation described by Hench [9].

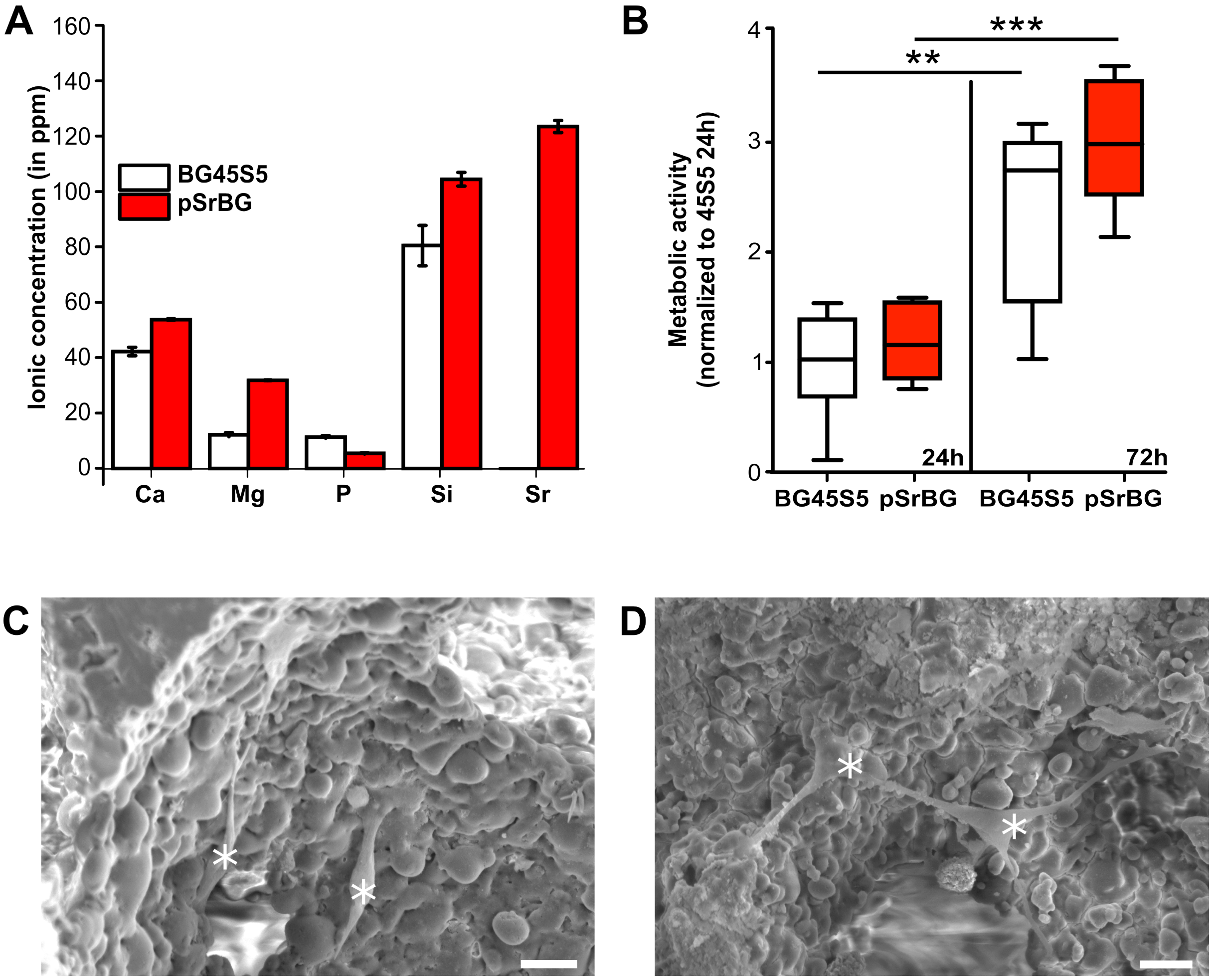

Figure S3. pSrBG displays a good biocompatibility. (A) Concentration of phosphorous, calcium, silicon and strontium (in ppm) in the BG45S5- and pSrBG-liquid extracts used to test the scaffold cytotoxicity as measured by ICP-OES. BG were incubated in cell culture medium (0.2 g/ml) for 24 hours at 37 °C and then removed by filtration. The partial dissolution of the BG into cell culture medium results in a modification of the ionic content of the medium that is dependent on the BG composition and particle size (surface area). **(B)** Relative metabolic activity measurement of MC3T3 osteoblasts treated with BG45S5- or pSrBG- liquid extracts for 24 hours and 72 hours ($n = 6$), showing that BG45S5 and pSrBG display a similar biocompatibility. **(C, D)** Cross-section SEM images of hMSC (asterisks) onto pSrBG scaffold after 7 days. Scale bars are 20 μm. The box plots represent the 5th to 95th percentiles, where the whiskers denote the maximum and minimum values. Asterisks denote statistically significant differences between indicated groups (**$p < 0.01$, ***$p < 0.001$).

Table S1. Composition of pSrBG defined by XRF. Table of the mol% of each oxide from the nominal composition and the composition obtained from the glass powder and scaffolds from XRF.

	SiO_2	Na_2O	CaO	SrO	K_2O	MgO	P_2O_5
Nominal composition (mol %)	44.5	4.0	17.8	17.8	4.0	7.5	4.5
XRF glass powder (mol %)	44.7	4.0	18.0	17.5	4.1	7.2	4.5
XRF pSrBG scaffold (mol %)	44.8	4.0	18.0	17.4	4.1	7.2	4.5

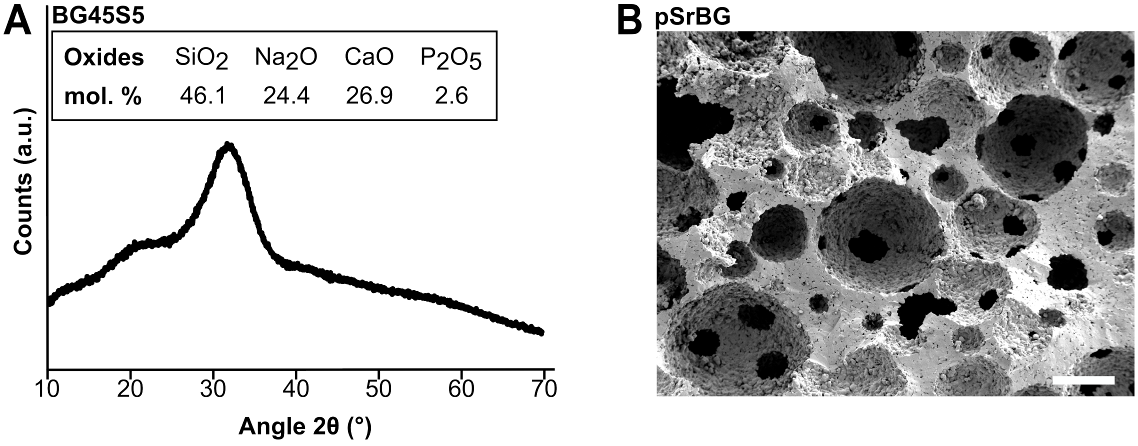

Figure S4: Material characterization. (A) XRD pattern of BG45S5 glass as produced. (B) Representative SEM image of pSrBG cross-section that shows the presence of pores and interconnects within the scaffold. The scale bar is 200 µm.

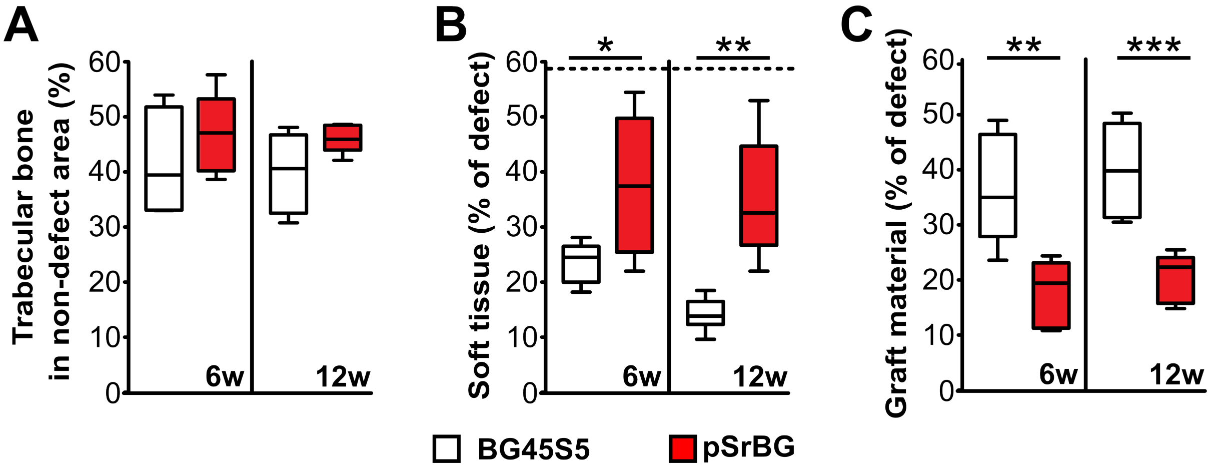

Figure S5. Histomorphometric analysis of native trabecular bone away from the defect areas and percentages of soft tissue and bone substitute material in defect areas. (A) Percentage of trabecular bone in the bone away from the bone defects treated with BG4S5 (n = 4 at week 6; and n = 5 at week 12) and pSrBG (n = 5 at both time points) to serve as a reference of the physiological amount of trabecular bone tissue. **(B)** Percentage of soft tissue within the BG45S5 and pSrBG (n = 5 at 6 weeks and n = 6 at 12 weeks for both materials) treated defects. **(C)** Percentage of graft within the defects (n = 5 at 6 weeks and n = 6 at 12 weeks for both materials). The box plots represent the 5th to 95th percentiles. Asterisks denote statistically significant differences between indicated groups (*$p < 0.05$, **$p < 0.01$, ***$p < 0.001$).

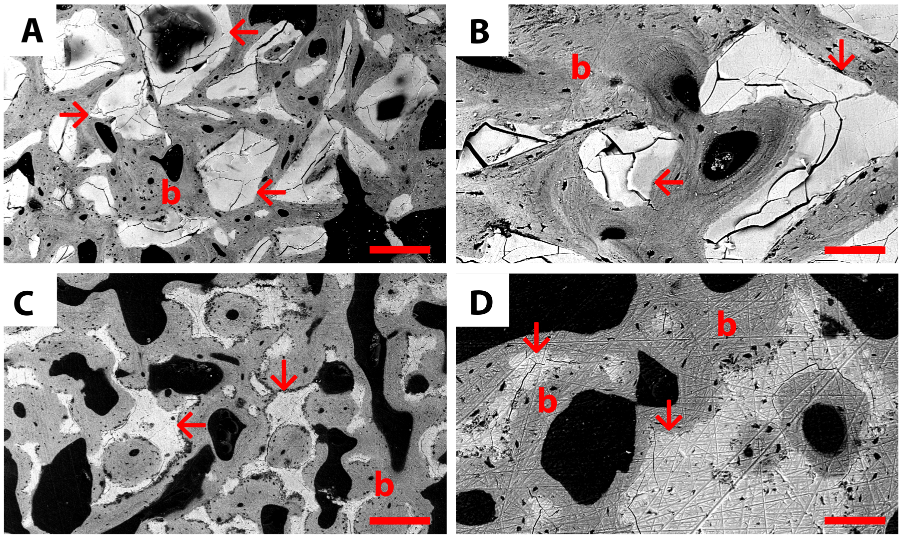

Figure S6. pSrBG induces the formation of well-organized lamellar bone tissue. Backscattered SEM images after week 12 of implantation of BG45S5 (A, B) and pSrBG (C, D). Arrows point at the remaining grafts; b represents new-formed bone. Scale bars in A, C are 270 μm; scale bars in B, D are 80 μm.

Table S2: Distance of lamellar and woven bone areas measured at the interface with the pSrBG or BG45S5 scaffold by FIB-SEM cross-section after 12 weeks of implantation. Cement plane delimited bone deposits of different generations or indicated that a remodeling event has already taken place. Three independent sections (from independent animals) were analyzed for each scaffold treatment.

Sample	Section	Lamellar bone	Woven bone	Undefined	Cement plane
**pSrBG**	Section 1-1	30 µm (79%)	-	8 µm	-
	Section 1-2	40 µm (100%)	-	-	-
	Section 1-3	45 µm (100%)	-	-	-
	Section 2-1	35 µm (50%)	35 µm (50%)	-	-
	Section 2-2	30 µm (100%)	-	-	-
	Section 3-1	20 µm (100%)	-	-	-
	Section 3-2	50 µm (77%)	-	15 µm	-
	Section 3-3	60 µm (80%)	-	15 µm	-
**Total sections (~400 µm)**		**81%**	**9%**	**10%**	
**BG45S5**	Section 1-1	10 µm (100%)	-	-	-
	Section 1-2	10 µm (22%)	35 µm (78%)	-	+
	Section 1-3	-	-	15 µm	?
	Section 2-1	30 µm (50%)	30 µm (50%)	-	+
	Section 2-2	30 µm (55%)	25 µm (45%)	-	-
	Section 3-1	20 µm (44%)	25 µm (56%)	-	+
	Section 2-2	60 µm (55%)	50 µm (45%)	-	+
**Total sections (~350 µm)**		**47%**	**49%**	**4%**	

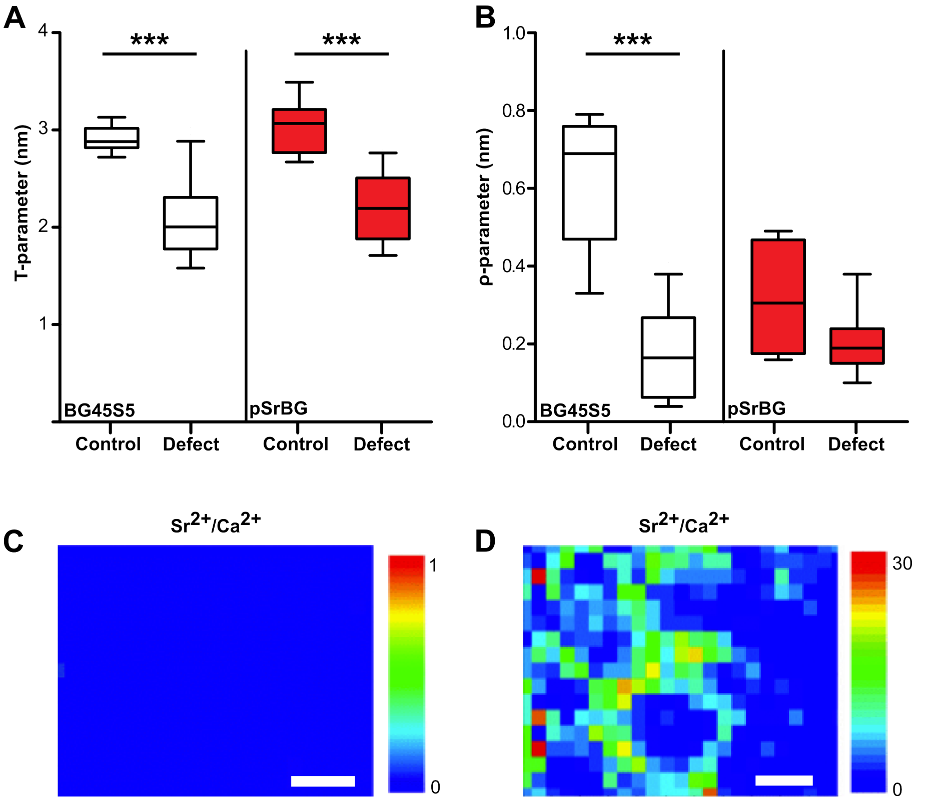

Figure S7. Quantification of T- and ρ- parameters, heat map of strontium ion to calcium ion ratio in native bone and treated defects. (A) Quantification of T-parameter as measured by SAXS, indicating significantly lower mean mineral crystal thickness in the ROI than in the control region in both groups. **(B)** Quantification of ρ-parameter as measured by SAXS, indicating significantly lower degree of alignment of the mineral crystals in the ROI than in the control region in the BG45S5 group. The box plots represent the 5th to 95th percentiles of values collected from 1 sample and are representative of 3 independent sections for each condition at 12 weeks. Asterisks denote statistically significant differences, calculated using 1-way ANOVA, between the control regions and ROI for each group (***p < 0.001). No significant differences were found when comparing pSrBG and BG45S5 defect regions. **(C-D)** Heat map of strontium ion to calcium ion ratio after 12 weeks of implantation in (C) BG45S5 and (D) pSrBG. Scale bars in C and D are 200 µm.

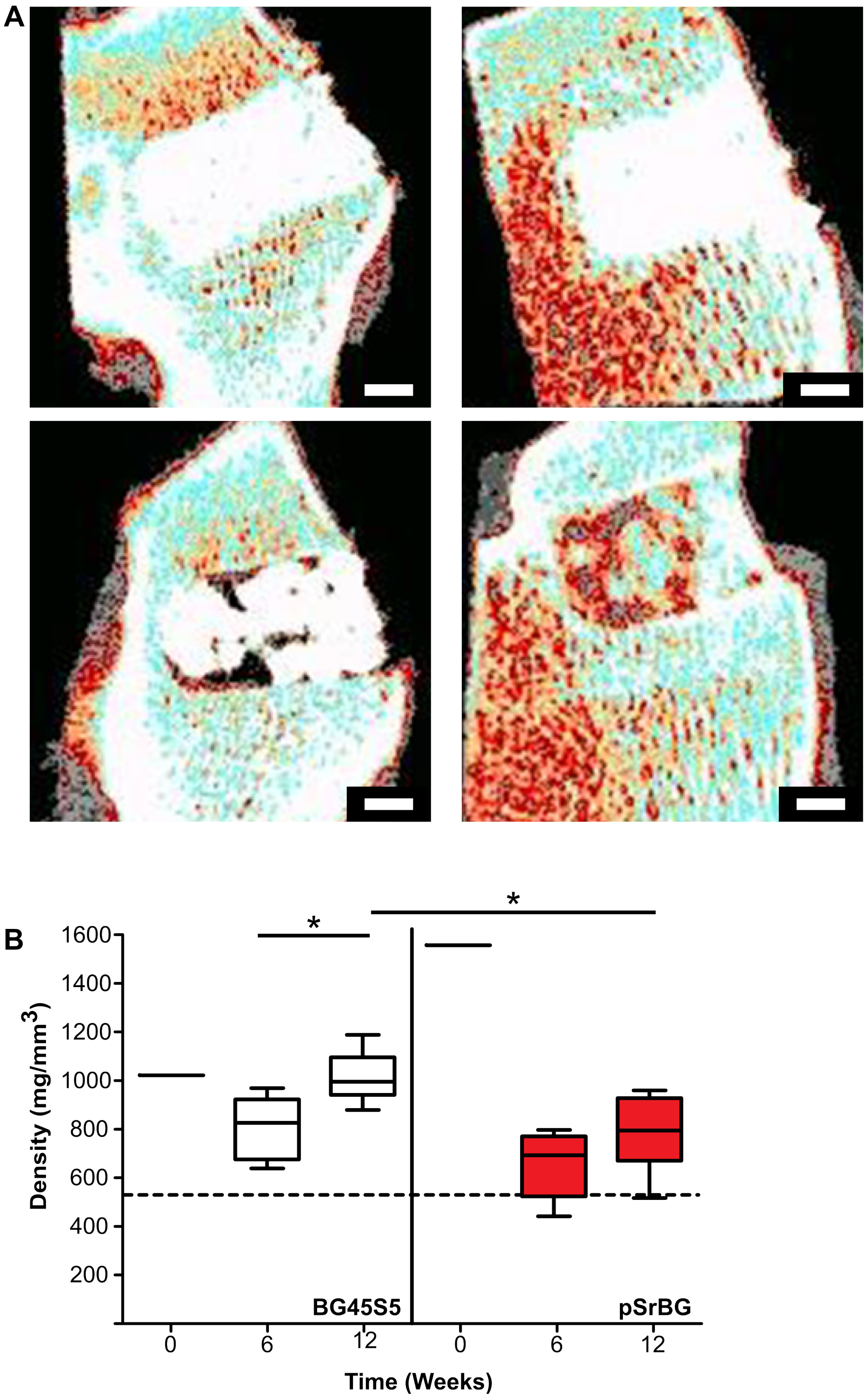

Figure S8. Density of pSrBG- and BG45S5-treated defects. (A) pQCT imaging showing the mineral density of BG45S5 (top row) and pSrBG (bottom row) at $t = 0$ (left column) and after 12 weeks after implantation (right column). Higher mineral density areas are in red. **(B)** Quantification of pQCT at different time points ($n = 1$ at week 0 both groups; $n = 6$ at weeks 6 and 12 for both groups) (*$p < 0.05$). The dashed line represents the median of mineral density of non-defect areas (obtained from 70 measurements). Strontium ions display a higher X-ray density than calcium because strontium's atomic number is higher, which explains a higher density in pSrBG-treated defects at day 0 when compared to BG45S5-treated specimens. Scale bars are 3 mm.

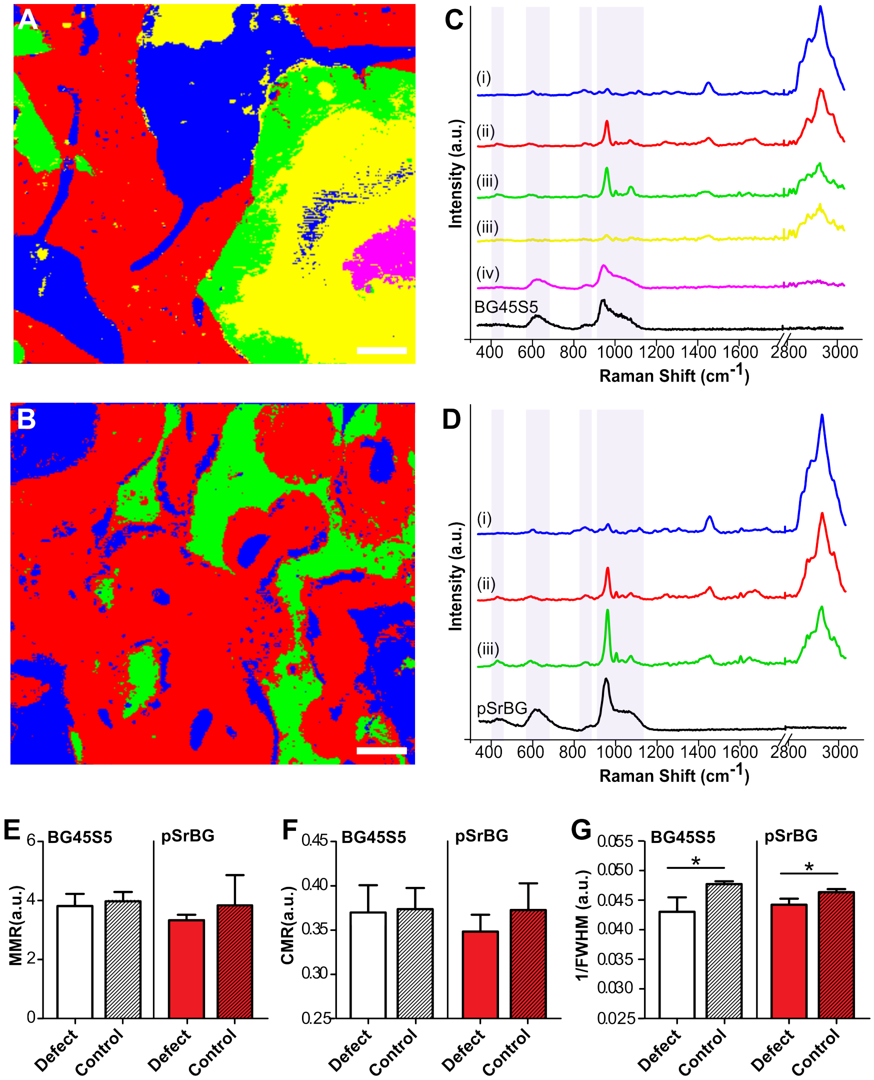

Figure S9. pSrBG promotes neo-bone with a native-like biochemical signature. **(A-D)** Distribution (A-B) and signature (C-D) of the characteristic Raman spectra identified by k-means clustering analysis of (A, C) BG45S5- and (B, D) pSrBG- treated defects. Raman spectral signatures (C-D) represent the (i) soft tissue, (ii) bone, and (iii) synthetic graft after modification in contact with biological fluids and (iv) synthetic graft with similar characteristics as the non-implanted produced BG. As produced, pSrBG and BG45S5 are shown for reference (black lines). **(E-G)** Raman spectroscopic evidences of the mineral-to-matrix ratio (MMR), carbonate-to-mineral ratio (CMR) and mineral crystallinity in native bone and treated defects. **(E)** Quantification of the MMR, as defined by the ratio between the phosphate 960 cm^{-1} band and the sum of the ~854 cm^{-1} and ~871 cm^{-1} proline and hydroxyproline bands. **(F)** Quantification of the CMR represented by the ratio of the $\nu_1CO_3^{2-}$ band at 1070 cm^{-1} and the 960 cm^{-1} band. **(G)** Quantification of the mineral crystallinity, determined by calculating the 1/half full width maximum of the 960 cm^{-1} peak. Data are expressed as mean ± SD of the median values of each individual section. For each section, median values were determined from ≥ 2 mapped regions per condition. Results are obtained from 3 independent sections (from independent animals). Asterisks denote statistically significant differences (*p < 0.05), calculated using an unpaired *t*-test, between the newly-formed bone and the control trabecular bone for BG45S5- and pSrBG- treated defects at 12 weeks (n = 3). Scale bars are 100 μm.

Supplemental references

[1] T. B. Kim, S. Yue, Z. Zhang, E. Jones, J. R. Jones, and P. D. Lee, 'Additive manufactured porous titanium structures: Through-process quantification of pore and strut networks', *J. Mater. Process. Technol.*, vol. 214, no. 11, pp. 2706–2715, 2014.

[2] T. Kokubo *et al.*, 'Ca,P-rich layer formed on high-strength bioactive glass-ceramic A-W', *J. Biomed. Mater. Res.*, vol. 24, no. 3, pp. 331–343, 1990.

[3] A. L. B. Maçon *et al.*, 'A unified in vitro evaluation for apatite-forming ability of bioactive glasses and their variants', *J. Mater. Sci. Mater. Med.*, vol. 26, no. 2, p. 115, 2015.

[4] P. Fratzl, N. Fratzl-Zelman, K. Klaushofer, G. Vogl, and K. Koller, 'Nucleation and growth of mineral crystals in bone studied by small-angle X-ray scattering', *Calcif. Tissue Int.*, vol. 48, no. 6, pp. 407–413, 1991.

[5] S. Rinnerthaler, P. Roschger, H. F. Jakob, A. Nader, K. Klaushofer, and P. Fratzl, 'Scanning small angle X-ray scattering analysis of human bone sections', *Calcif. Tissue Int.*, vol. 64, no. 5, pp. 422–429, 1999.

[6] P. Fratzl, S. Schreiber, and K. Klaushofer, 'Bone mineralization as studied by small-angle x-ray scattering', *Connect. Tissue Res.*, vol. 34, no. 4, pp. 247–254, 1996.

[7] M. J. Coathup, Q. Cai, C. Campion, T. Buckland, and G. W. Blunn, 'The effect of particle size on the osteointegration of injectable silicate-substituted calcium phosphate bone substitute materials', *J. Biomed. Mater. Res. B Appl. Biomater.*, vol. 101B, no. 6, pp. 902–910, 2013.

[8] D. Sriranganathan, N. Kanwal, K. A. Hing, and R. G. Hill, 'Strontium substituted bioactive glasses for tissue engineered scaffolds: the importance of octacalcium phosphate', *J. Mater. Sci. Mater. Med.*, vol. 27, 2016.

[9] L. L. Hench, R. J. Splinter, W. C. Allen, and T. K. Greenlee, 'Bonding mechanisms at the interface of ceramic prosthetic materials', *J. Biomed. Mater. Res.*, vol. 5, no. 6, pp. 117–141, 1971.
